# A review on thermoresponsive PNIPAM hydrogels for regenerative medicine: from mechanical design to tissue adhesion

**DOI:** 10.1093/rb/rbag045

**Published:** 2026-03-16

**Authors:** Aaima Siddiqui, Muhammad Touqeer, Farjana Akter Ritu, Muhammad Adnan Haider, Akhlaq Ahmed, Oscar Senanu James-Ocloo, Naseer Ullah, Longfei Wang, Jian Zeng, Longsheng Gao, Di Huang

**Affiliations:** Department of Biomedical Engineering, Research Center for Nano-Biomaterials & Regenerative Medicine, Shanxi Key Laboratory of Functional Proteins, College of Artificial Intelligence, Taiyuan University of Technology, Taiyuan 030024, China; Institute of Biomedical Engineering, Shanxi Key Laboratory of Materials Strength & Structural Impact, Taiyuan University of Technology, Taiyuan 030024, China; Department of Biomedical Engineering, Research Center for Nano-Biomaterials & Regenerative Medicine, Shanxi Key Laboratory of Functional Proteins, College of Artificial Intelligence, Taiyuan University of Technology, Taiyuan 030024, China; Institute of Biomedical Engineering, Shanxi Key Laboratory of Materials Strength & Structural Impact, Taiyuan University of Technology, Taiyuan 030024, China; Department of Biomedical Engineering, Research Center for Nano-Biomaterials & Regenerative Medicine, Shanxi Key Laboratory of Functional Proteins, College of Artificial Intelligence, Taiyuan University of Technology, Taiyuan 030024, China; Institute of Biomedical Engineering, Shanxi Key Laboratory of Materials Strength & Structural Impact, Taiyuan University of Technology, Taiyuan 030024, China; Department of Biomedical Engineering, Research Center for Nano-Biomaterials & Regenerative Medicine, Shanxi Key Laboratory of Functional Proteins, College of Artificial Intelligence, Taiyuan University of Technology, Taiyuan 030024, China; Institute of Biomedical Engineering, Shanxi Key Laboratory of Materials Strength & Structural Impact, Taiyuan University of Technology, Taiyuan 030024, China; Department of Biomedical Engineering, Research Center for Nano-Biomaterials & Regenerative Medicine, Shanxi Key Laboratory of Functional Proteins, College of Artificial Intelligence, Taiyuan University of Technology, Taiyuan 030024, China; Institute of Biomedical Engineering, Shanxi Key Laboratory of Materials Strength & Structural Impact, Taiyuan University of Technology, Taiyuan 030024, China; Department of Biomedical Engineering, Research Center for Nano-Biomaterials & Regenerative Medicine, Shanxi Key Laboratory of Functional Proteins, College of Artificial Intelligence, Taiyuan University of Technology, Taiyuan 030024, China; Institute of Biomedical Engineering, Shanxi Key Laboratory of Materials Strength & Structural Impact, Taiyuan University of Technology, Taiyuan 030024, China; Department of Biomedical Engineering, Research Center for Nano-Biomaterials & Regenerative Medicine, Shanxi Key Laboratory of Functional Proteins, College of Artificial Intelligence, Taiyuan University of Technology, Taiyuan 030024, China; Institute of Biomedical Engineering, Shanxi Key Laboratory of Materials Strength & Structural Impact, Taiyuan University of Technology, Taiyuan 030024, China; Department of Biomedical Engineering, Research Center for Nano-Biomaterials & Regenerative Medicine, Shanxi Key Laboratory of Functional Proteins, College of Artificial Intelligence, Taiyuan University of Technology, Taiyuan 030024, China; Institute of Biomedical Engineering, Shanxi Key Laboratory of Materials Strength & Structural Impact, Taiyuan University of Technology, Taiyuan 030024, China; Shanxi Academy of Eco-Environmental Planning and Technology, Taiyuan 030024, China; Shanxi Academy of Eco-Environmental Planning and Technology, Taiyuan 030024, China; Department of Biomedical Engineering, Research Center for Nano-Biomaterials & Regenerative Medicine, Shanxi Key Laboratory of Functional Proteins, College of Artificial Intelligence, Taiyuan University of Technology, Taiyuan 030024, China; Institute of Biomedical Engineering, Shanxi Key Laboratory of Materials Strength & Structural Impact, Taiyuan University of Technology, Taiyuan 030024, China

**Keywords:** PNIPAM, hydrogel dressing, wet environment, mechanical properties, adhesion

## Abstract

Poly(N-isopropylacrylamide) (PNIPAM) hydrogels are potential wound dressings due to their lower critical solution temperature (LCST) of 32°C, close to physiological temperature, enabling injectable or spreadable formulations that quickly form a gel on irregular wounds while withstanding tissue motion. These systems aim to balance skin-like mechanical integrity, strong dry and wet adhesion and deliver stability after placement. This review summarizes the impact of the LCST driven phase transition on water content, pore structure, stiffness and interfacial hydration and how these effects regulate adhesion strength and failure mode. It compares major mechanical reinforcement strategies in PNIPAM-based dressings including hybrid nanocomposites, double network architectures, dynamic or sacrificial crosslinking strategies that enhance toughness, stretchability and recovery. It also describes multifunctional systems incorporating antibacterial activity, hemostasis, sensing, photothermal response or controlled drug release with emphasis on their effect on mechanics and adhesion. Since adhesion in soft hydrogels is strongly correlated with viscoelastic behavior, the review identifies rheological measures (*G*′/*G*″ trends, shear thinning, yield behavior and recovery) as measures of injectability and wet adhesion stability. Lastly, essential translational barriers are defined, including limited biodegradability, additive cytotoxicity, sterilization, durability in exudate and motions and standardized testing and reporting to facilitate reliable comparison and clinical relevance.

## Graphical Abstract

**Graphical Abstract:**
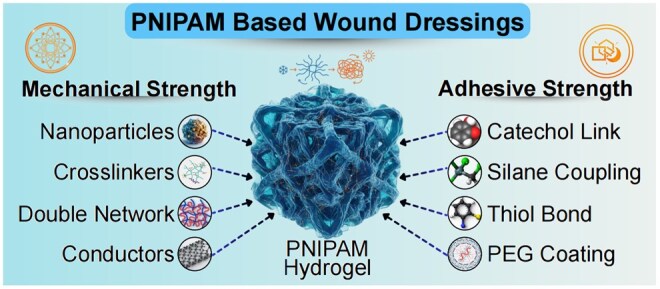

## Introduction

A significant barrier to healing of wounds is mechanical incompatibility between synthetic wound dressings and the native tissue. Dressings that lack the ability to mimic the compliance and deformability of skin may produce discomfort, disrupt conformal contact and offer inadequate biomechanical assistance to cell proliferation and tissue healing [1, 2]. These variations can slow the healing process and lower the overall treatment effectiveness, and it is important to note that wound dressings that better mimic the mechanical performance of native tissue are needed [3]. Hydrogels with unique 3D network structures have become the focus of modern biomedical practice because of their ability to take large volumes of water and simultaneously maintain mechanical stability [4]. They have built-in flexibility and can so far respond to a variety of biomedical issues such as tissue engineering, bioelectronics, drug delivery and wound management. The characteristics that they possess make them specifically competitive in the treatment of chronic wounds. Hydrogels also support the healing process, since they support moisture state, therefore, preventing crust formation—an essential part of the tissue regeneration process [5]. Their biomimetic porosity provides the right microenvironmental healing, and their self-healing qualities allow the adherence to irregular wound beds, which maintains constant contact and protection of the infected site [6]. Moreover, they serve as the obstacle to the invasion of bacteria that significantly decreases the risk of infection in nonhealing wounds [5, 7].

More recent development in hydrogel design has produced injectable self-healing formulations, enabling the material to be shaped into various wound geometries and have sufficient mechanical strength [8, 9]. Their adjustable mechanical characteristics enable them to mimic natural tissues extensively, thus, improving integration and functionality during the healing process [10]. The versatility of the smart hydrogel makes it a better replacement of traditional wound dressing. Minimally invasively, thermoresponsive sol to gel transition hydrogels can be used to regenerate tissues [11]. Hydrogel wound dressings are highly humidity sensitive in their mechanical properties and tend to be softened in humid environments and hardened in dry ones. Experimental research has developed some predictable patterns of swelling behavior versus elastic modulus, such that one can accurately predict stiffness when the humidity varies [12]. Advances in environmentally adaptable hydrogels have also augmented their ability to retain wet, soft properties under highly harsh conditions to expand clinical utility [12, 13].

Successful wound closure and minimally invasive procedures are entirely based on effective tissue adhesion. Sufficient compliance with adhesive covers wound healing, relieves pain and accelerates wound healing by maintaining proper positioning on the tissue, leading to minimal motions and irritation at the wound area [14]. The bilateral relationship between mechanical behavior, hydration and tissue adhesion in hydrogel wound dressing has a huge impact on their curative performance. Unless the hydrogel can strongly adhere to the tissue, robust tissue adhesion is necessary to ensure that it stays in place [15], and that mechanical toughness and extensibility are necessary to allow the hydrogel to adapt to dynamic movements of tissues [16]. Another critical factor is hydration because a damp environment is known to speed up healing in wounds by increasing cellular growth and mitigating the risk of infection [17]. However, mechanical strength and adhesion may be affected by excess hydration, and therefore, a carefully balanced hydrogel network is required [18]. Overall, the effective design of hydrogel wound dressings must maintain a balanced approach to tissue adhesion, mechanical properties and hydration to promote successful wound healing yet satisfy physiological and mechanical factors of the wound environment.

Poly (N-isopropylacrylamide) (PNIPAM) is one of the most popular stimuli-responsive polymers that have been studied to develop advanced wound dressings. PNIPAM has a lower critical solution temperature (LCST) of about 32°C. This strong thermoresponsive characteristic of PNIPAM-based hydrogels makes them particularly useful in biomedical research because their volume and properties can be precisely manipulated using external thermal stimuli [19]. This property is also utilized in composite hydrogels, where the PNIPAM is mixed with other polymers or nanoparticles to improve mechanical strength, biocompatibility and biodegradability, which deal with inherent drawbacks of pure PNIPAM hydrogels [20]. The outstanding mechanical strength and high elongation at break are a highly beneficial strength of PNIPAM-based hydrogels, and this quality is obligatory in preserving structural integrity and the relevant performance of dressing [21]. To a great extent, the adhesive behavior of hydrogels based on PNIPAM is explained by the ability of the compound to form hydrogen bonds and hydrophobic interactions with biological tissues in association with other biopolymers, which is a very important attribute of this compound to maintain the use of dressing and to promote healing [22].

PNIPAM-based dressings have adjustable contraction and adhesion, self-shrinkage and can adjust the rate of shrinkage to support quick wound healing without using sutures [23]. Additionally, controlled drug release properties into PNIPAM hydrogels allow thermo-regulated loading and release of therapeutics, and thus, make it possible to customize antimicrobial efficacy and overcome potential infection risks in hydrated wound environments [24, 25]. Therefore, the general functionality and efficacy of PNIPAM-based dressings in managing wounds rely on the prudent consistency between their hydrophilic and hydrophobic attributes. All these combined features make PNIPAM-based hydrogels incredibly strong in wound dressing, providing a versatile platform which facilitates wound healing, prevents infection and promotes healing by providing controlled swelling, mechanical compliance and strong tissue adhesion [26].

The use of PNIPAM hydrogel, although having the above advantages, has some significant limitations, which must be taken into account during clinical trials. The most important of them is their low level of biodegradability, which predisposes to the chronic bioaccumulation in the host; this deficiency can be basically explained by the fact that PNIPAM is of synthetic origin and is resistant to degradation in physiological conditions [27]. Additionally, the thermal hysteresis of PNIPAM hydrogels during swollen to deswollen occurs with a large magnitude, hence, undermining thermal responsiveness and mechanical strength [28, 29]. In turn, overcoming these difficulties by means of informed material design and hybridization methods is one of the main priorities in the evolution of PNIPAM-based hydrogel wound dressings.

**Figure 1:**
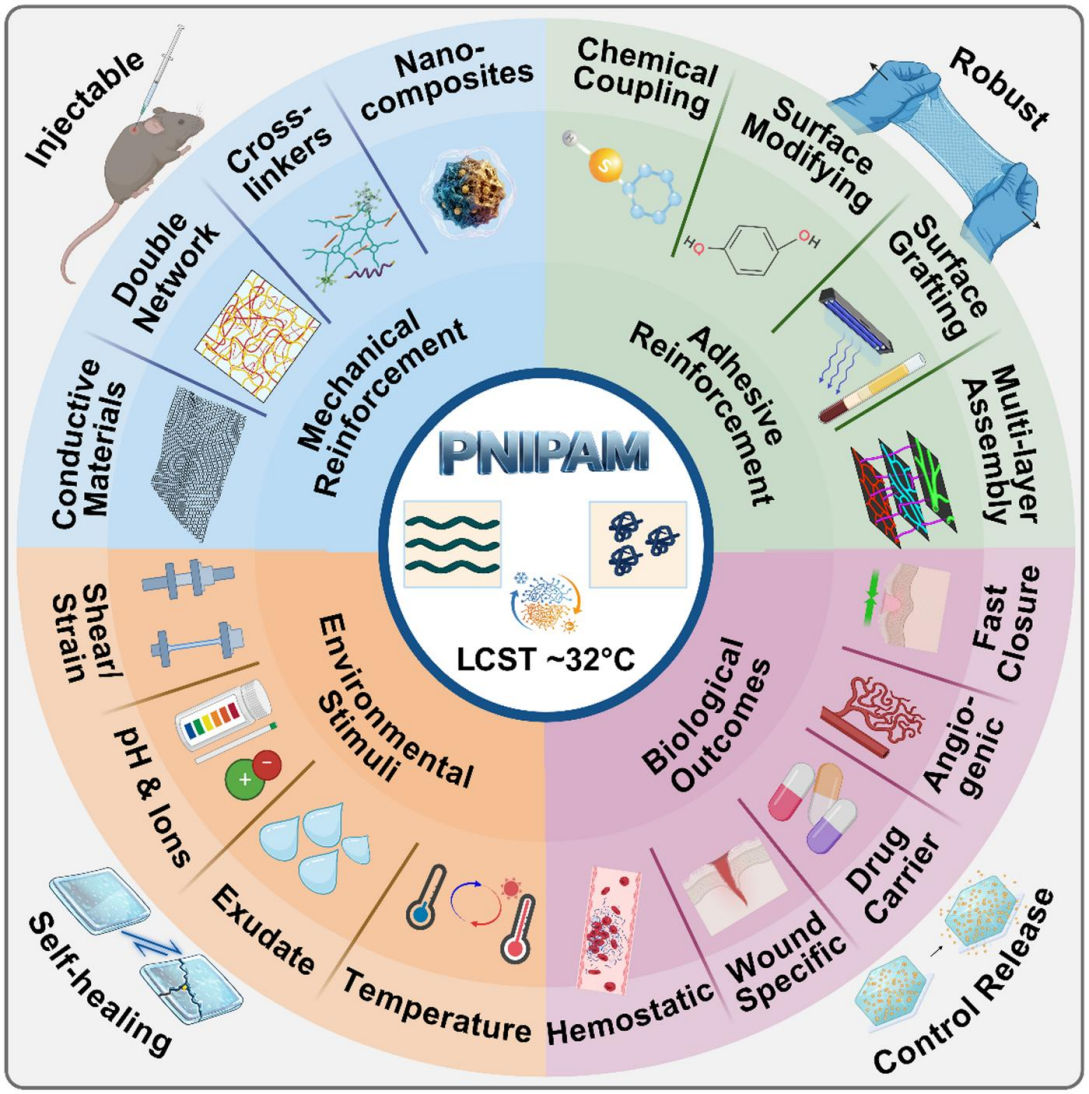
Integrated design framework for thermoresponsive PNIPAM wound dressings.

Mechanical and adhesive qualities of PNIPAM-based hydrogels are essential to examine to ascertain durability and the ability to be used in medical practice. This review focuses on the mechanical performance of PNIPAM-based dressings, with marked emphasis on stretching, bending and compressive resistance under physiologically relevant conditions. Figure 1 summarizes the logic of this review. Starting from PNIPAM’s LCST, we connect network and adhesion reinforcement approaches with wound-relevant stimuli and the resulting performance and healing outcomes. This framework is used as a roadmap for the subsequent sections on mechanics, adhesion and translational relevance.

## Thermoresponsive mechanism of PNIPAM hydrogels

Although the LCST behavior of PNIPAM is briefly presented in the previous section, a specific mechanistic framework is needed to explain how the operation of this thermally controlled phase transition dictates the ability to operate in wound settings. The coil-to-globule conversion induced by LCST changes the hydration of polymer, its structure and their interactions at interfaces and shrinks the molecular-scale events into macroscopic rises in stiffness, moisture responsiveness, adhesion and mass transport. This part summarizes these associations to explicitly connect PNIPAM thermoresponsive theory to performance results of wounds.

### LCST driven coil-globule transition

PNIPAM forms coil-to-globule alteration at the temperature beyond LCST and, thus, it loses its affinity to water and triggers the dehydration of the network. This conversion is identified by the fact that PNIPAM chains are compacted into more hydrophobic forms, which also drives out the molecules of water of the polymer matrix [30–32]. The dehydration mainly because of the destruction of the hydrogen bonds between the amide groups of PNIPAM and the surrounding water molecules; with increase in temperature, these bonds break whereas hydrophobic interactions between the isopropyl groups of PNIPAM increase [33]. This leads to a loss of solubility of the polymer in water and to the collapse of conformation of polymer, which forms the molecular basis of the LCST-mediated phase transition in PNIPAM hydrogels [33, 34].

As temperature reaches and exceeds the LCST, the coil-to-globule transition of PNIPAM chains proceeds via the aggregation of individual chains into compact globular chains, owing to the benefit of increased strength of the hydrophobic forces in the polymer network [34, 35]. The overall breakdown of these chains causes a macroscopic shrinkage of the network, also known as the network collapse, that occurs as a reduction in the mesh size and increased hydrogel densification [36]. The scale and acuity of this LCST-based network breakdown are influenced by the polymer structure and density of cross-linking and subsequently determine the way individual chain transitions are relayed through the bulk hydrogel deformation [37].

**Figure 2:**
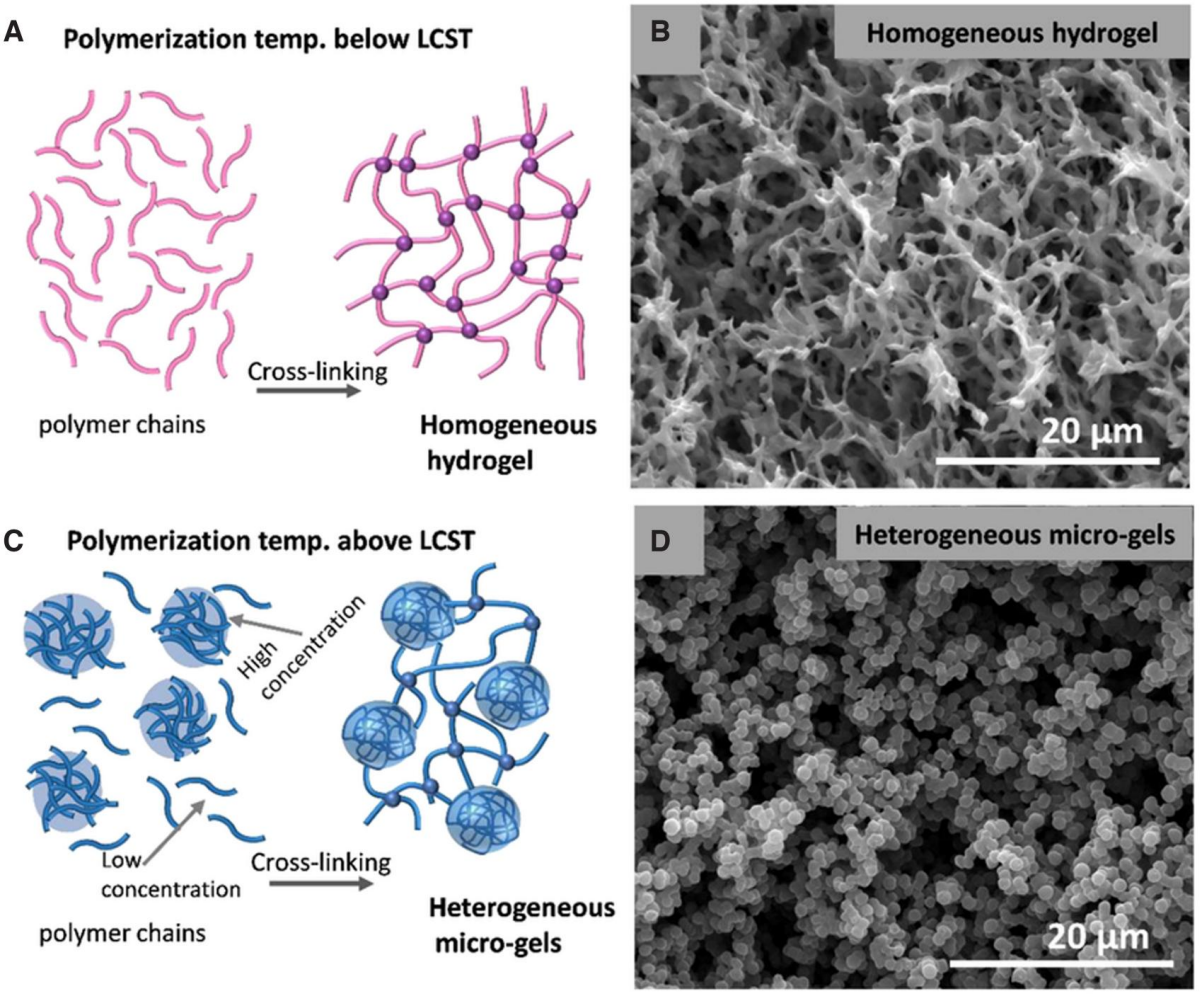
Thermoresponsibility of the PNIPAM hydrogel prepared with different temperatures. Scheme and SEM images of PNIPAM hydrogels obtained by polymerizing (**A, B**) below LCST and (**C, D**) above LCST. Schematic diagrams showing the gelation process at two preparation temperatures. Gelation at (**A**) lower and (**C**) higher temperatures than the LCST. Reproduced with permission [38], Copyright 2022, *ACS Sustainable Chemistry & Engineering*.

Figure 2 schematically depicts the effect of temperature on the assembly of PNIPAM networks compared to the LCST. Below the LCST results in homogeneous hydrogel network polymerization, and above the LCST results in chain collapse and phase separation, thus, forming heterogeneous microgel-based structures.

### Impact of phase transition on functional properties

The phase transition under LCST direct influence of mechanical, interfacial and transport performance of PNIPAM-based hydrogels, converting the events occurring in the molecular scale into macroscopic functional performance. PNIPAM starts to show a significant rise in mechanical properties, e.g. stiffness and increase in Young's modulus as the temperature nears and rises beyond the LCST-(or volume phase transition-temperature). This increase in modulus reduces the volume of hydrogel and involves polymer chains closer to each other, therefore, creating a stronger and more rigid network structure [39, 40]. As a result, the swollen condition under the LCST exhibits less rigidity and the collapsed condition over the LCST exhibits higher mechanical strength [40].

The PNIPAM surfaces above the LCST become more hydrophobic, changing both the wettability and the interfacial forces that play a pivotal role in transducing the subsequent tissue interaction and adhesion behavior [33, 41,42]. The PNIPAM hydrogels release water above the LCST due to thermally induced phase transition which changes polymer–water interactions. In addition to the LCST, the hydrophobic force between the chains of the PNIPAM is also predominant over the hydrophilic interaction between the polymer and surrounding water (Figure 2C and D) and chain collapse and subsequent decrease in the volume of the hydrogel occurs [36, 43]. Such a change of a hydrated, extended form to a dehydrated, collapsed form is coupled with hydrophobic aggregation of the chains forming part of the affected polymers which removes water of the network and is characterized by a strong deswelling effect [41]. In this way, the water expulsion under LCST represents a direct interaction between a phase transition involving a molecular scale and the macroscopic volumetric change in PNIPAM hydrogels [44].

Above LCST, the dehydration of the polymer causes the adhesive property to improve; conversely, heating below LCST restores the hydration, hence, reducing adhesion [45]. This two-way alteration allows the achievement of high adhesion (when it is in the collapsed state) and low residual adhesion (when it is in the swollen state), thus, forming the core principle of temperature-controlled adhesion switching in PNIPAM hydrogels [36].

The hydrophilic and swollen structure of PNIPAM hydrogel is maintained below LCST, which practically ensures the fixation of encapsulated drugs to the polymeric chain. When the hydrogel passes the LCST, it becomes hydrophobic and collapses its network, and entrapped drug molecules are released. This thermodynamically reversible phase transition has been widely applied in PNIPAM-based drug delivery systems to achieve temperature-controlled and targeted drug release [46].

### Relevance of LCST behavior in physiological environments

The LCST of PNIPAM, which is close to the physiological temperature, offers specific benefits to wound dressing use, via the capability of the hydrogel to react instantaneously to thermal environment of the skin and wound interface. This transition causes mechanical strengthening and conformability of the hydrogel at physiological temperature and allows adaptation of the hydrogel to wound geometry, maintaining contact with tissues over time [47]. In addition, thermoresponsibility of PNIPAM enables controlled release of drugs and control of moisture in the wound healing process. The phase change induced by LCST is beneficial to temperature-controlled release of drugs, which is beneficial in controlling the level of inflammation and promoting tissue repair and regeneration, and deswelling provides local cooling and anti-inflammatory benefits [48, 49]. Instead, a temperature lower than the LCST causes hydrogel to swell due to water reabsorption, which reduces the adhesive properties and allows the impact of the gel to be detached without suffering or harming the substrate below [50]. A combination of these properties highlights the clinical importance of PNIPAM hydrogels as smart wound dressings that would perform well in physiological environments [51].

Based on the LCST directed molecular and network level processes outlined above, the following section explores the further conversion of these thermoresponsive changes into measurable mechanical strength and tissue adhesion capabilities of PNIPAM-based hydrogels in wound-relevant conditions.

## PNIPAM-based hydrogel dressings for regenerative medicine

In the last decade, a lot of interest has been given to hydrogels regarding tissue adhesive and wound dressing proposals, which has been primarily attributed to their high-water composition, inherent biocompatibility and sensitivity to extrinsic stimuli [52, 53]. Empirical studies highlight the importance of the fact that a wound dressing hydrogel must meet several criteria at once to be the optimal one. First, mechanical properties such as adjustable adhesion and contractility are important, so that the hydrogel is able to conform to the wound site and allows scar-free healing through the reduced infiltration of inflammatory cells, collagen deposits as well as the self-healing properties [16]. Second, the material should have a high level of biocompatibility and assist in the growth and movement of cells, which can be evidenced by the hydrogel that can stimulate the growth of human endothelial cells and fibroblasts, thus, increasing angiogenesis and repair of the skin [54]. Third, the hydrogel must mimic the hierarchical architecture of extracellular skin matrix to demonstrate a suitable environment for wound healing [55]. Fourth, antimicrobial action is essential, especially in infected wounds; this may be implemented by use of antimicrobial agents to maintain antibacterial action and prevent inflammation [56].

Increased levels of reactive oxygen species within the wound microenvironment have been shown to impair the functionality of cells and delay tissue healing; thus, incorporating antioxidant functionality into the fabrication of hydrogel dressings has been suggested as a good approach to promote healing [57, 58]. Besides, hydrogel should be highly porous to allow the exchange of fluids and allow gases to pass through and at the same time, it should have the right swelling behavior to maintain moisture. The hydrogel should also offer excellent drug delivery properties and release bioactive substances to the wounding environment, enhancing healing [59]. The hydrogel should also have the ability to remodel the immune microenvironment of wounds, including diabetic foot ulcers (DFUs), to repair the damaged tissue [60]. Altogether, these properties make hydrogels a very appealing option as the next-generation type of wound dressing, giving them excellent therapeutic potential in a wide range of pathologies. Hydrogels made of PNIPAM have better performance in wound care. These stimulus responsive systems have unique physicochemical characteristics, such as temperature responsive volumetric control, adjustable mechanical behavior and considerable adhesion ability on hydrophilic and hydrophobic surfaces.

Besides mechanical properties, PNIPAM-hydrogels are also characterized by an adhesion capacity that is depended on. The dressing is able to stick to both the dry and wet surfaces using this property such that the coverage is retained even with the presence of wound exudates or ambient moisture.

Moisture is critical to the behavior and performance of PNIPAM-based hydrogel dressings. The presence of moisture influences both the swelling and deswelling dynamics and the polymer chain dynamics of these hydrogels [61]. In addition, moisture concentration will determine the dehydration dynamics of hydrogel wound dressing in which the rate of dehydration is negatively proportional to the thickness of the dressing and less dependent on the original water content [62]. PNIPAM/Polypyrrole (PNIPAM/Ppy) hydrogels have high hygroscopic efficiency, with up to 80% moisture capacity under different humidity conditions, and temperature has no significant influence on hygroscopically functioning below 40°C temperature [63]. Hydrogel dressings can even mimic the effect of perspiration that happens on skin, and this can be used to affect surface characteristics, including friction and thermal conductivity, making them useful in preventing injuries related to devices [64].

Table 1 provides a summary of the adaptive behavior of the PNIPAM-based hydrogel dressings to different moisture levels, which shows that they can control their mechanical behavior and still be used successfully in dry and wet conditions.

**Table 1:** Moisture sensitivity and adaptability of PNIPAM-based hydrogel dressings in different environments.

Moisture condition	Observed behavior	Functional impact on dressing	References
High humidity (wet tissue)	Swelling, softening of matrix, enhanced water uptake	Improves comfort, conformability and wound hydration	[63, 65]
Low humidity (dry skin)	Stiffening and reduced chain mobility	Enhances support and structure retention	[12, 13]
Humidity cycling	Reversible swelling/deswelling and modulus adaptation	Suitable for dynamic environments and long-term wearability	[66, 67]
Perspiration-like effect	Mimics skin’s moisture regulation and thermal conductivity	Reduces friction, prevents pressure sores and device-related injuries	[64]
Variable wound exudate	Adjusts dehydration rate depending on thickness and water content	Helps control fluid balance in acute and chronic wounds	[62, 63]

The purpose of this review is to conduct a systematic review of recent developments in the domain of the mechanical viability of PNIPAM-based hydrogel dressings in terms of tissue adhesion in dry and moist conditions. Besides, it will explain how the density of crosslink, compositions of polymer and environmental stimuli jointly affect the mechanical properties of the PNIPAM-based hydrogel. Lastly, the article will discuss the methods that have been employed to increase the mechanical strength as well as tissue adhesion of PNIPAM-derived hydrogels used as wound dressings.

## Mechanical design principles of PNIPAM-based hydrogels for regenerative medicine

### Mechanical requirements

To effectively match skin and soft tissue, hydrogel wound dressings need to exhibit a balanced mix of mechanical strength, elasticity, toughness and interfacial stability [68]. They must have suitable elastic modulus and tensile strength that help to provide structural integrity without mechanical incompatibility with the surrounding tissue. Reported hydrogel systems have compressive strengths comparable to natural skin (about 0.3–1.4 MPa), tensile properties that are a few tens to hundreds of kilopascals, meaning that they provide sufficient mechanical support without excessive rigidity [66, 68–70]. High stretchability and elongation at break are specifically vital in dressings on dynamic parts of the body because they enable a conformal deformation with the movement of the joint and counteracts mechanical irritation [16]. In addition, mechanical performance also depends on fatigue resistance and self-healing properties necessary to maintain mechanical performance with repeated loading and long-term usage [16, 71].

Hydrogel wound dressings should also be mechanically stable in high water contents to absorb wound exudates without loss of structural integrity. Also, tissue adhesion and shear resistance ensure stable fixation in wet conditions, eliminating delamination during the healing process. Overall, hydrogels with a moderate stiffness, good stretchability, toughness, fatigue resistance and durable adhesive properties are the most suitable to replicate the mechanical behavior of the skin and soft tissue, and thus, provide a mechanically supportive environment during wound healing [68, 72].

Effective wound dressing hydrogels exhibit a wide but application-relevant range of mechanical properties. Elastic moduli have been reported to range from soft, skin-like hydrogels (from 10 to 20 kPa) to mechanically strong systems with values reaching 100 MPa, depending on structural design and application [73, 74]. Stretchability can usually range between 300% and 3000%, allowing conformal deformation of dynamic tissues without mechanical failure. The values of toughness vary from several tens of kJm^−3^ to several hundreds of kJm^−3^ (up to 0.5 MJm^−3^), indicating the effectiveness of energy-dissipation in toughened hydrogel networks [74, 75].

Hydrogel dressings on dynamic tissues require fatigue resistance since the skin and other soft tissues are subjected to continuous cyclical deformation during movement, which may cause progressive mechanical damage and functional loss [23, 76]. Hydrogels that are not fatigue resistant can undergo crack initiation, delamination or rupture during repeated loading, and hence, reduce wound coverage and healing effectiveness [76]. Viscoelastic and toughened hydrogel systems dissipate mechanical energy in a better way and maintain structural integrity in cyclic stress conditions, thus, retaining adhesion and mechanical support during long-term usage [77]. Overall, adequate fatigue resistance guarantees long lasting mechanical functioning, consistent wound care and enhanced healing results in the mechanically active areas [23, 76, 77].

### Energy dissipation and toughening mechanisms

The mechanical toughness of hydrogel networks is largely explained by their ability to dissipate mechanical energy through a collection of complementary mechanisms, rather than the storage of elastic energy [78–81]. One such mechanism is the use of dynamic and reversible cross-linking motifs, including ionic contacts and hydrogen bonds, that, when stressed under a mechanical force, dissociate to release energy and, conversely, re-associate, thus, preventing disastrous fracture [78–80].

In addition, dual-and multi-network structures add toughness to the structure by the incorporation of sacrificial components that dispel energy preferentially, preserving the structural integrity of the main, load-bearing network [81]. These mentioned processes enable hydrogels to receive large amounts of energy through bond breaking, viscoelastic hysteresis and network reconfiguration, achieving high stretchability, fracture resistance and mechanical stability in repetitive deformation [78–81].

Sacrificial bonds help to increase fracture resistance of hydrogels by preferential rupturing when subjected to mechanical stress, thereby absorbing the applied energy before the damage is propagated to the primary polymer network [82]. This controlled bond rupture works as an effective energy dissipation method, which reduces concentration of stress at crack tips and delays cracks initiation and growth [83]. Since many sacrificial interactions are reversible, such as hydrogen bonding, the broken bonds can be reformed after deformation, and thus, this allows repeated energy loss cycles without permanent network failure [84].

In hydrogels with micelle-assisted chain alignment, sacrificial interactions help the polymers to slide over each other and the redistribution of stress to increase the impact resistance and overall mechanical durability [82]. Hydrophobic domains and semicrystalline regions also play a role in toughness by combining network stiffening and energy dissipation through entanglements during large deformation [85]. Strong but reversible hydrogen-bonding motifs, including covalent-like hydrogen bonds, provide further fracture resistance by maintaining network integrity while continuously dissipating mechanical energy [86].

Viscoelastic hysteresis is an important contributor to the toughness of hydrogels as it dissipates mechanical energy during cyclic deformation due to rearrangement of the polymer chains with time. This dissipation of energy in bulk form, in the form of stress–strain hysteresis allows hydrogels to absorb energy applied beyond their intrinsic fracture energy, augmenting the resistance to mechanical failure [87]. Reversible physical crosslinks allow repeated bond rupture and reformation under load, providing an ongoing dissipation of energy during deformation [88]. Polymer network designs that create more entanglement in the chains or limit slippage of the segments further control the hysteresis behavior and enhance the mechanical stability [89].

As the localization of deformation occurs and initiation of microcracks take place, energy dissipation switches from bulk hysteresis to fracture controlled mechanisms. Crack deflection improves the toughness by changing the crack propagation path, and therefore, reduces the concentration of stress at the crack tip and augment the energy consumption for fracture [83]. In polymer hydrogels with interpenetrating structure or anisotropic network structure, cracks are forced to follow tortuous paths to promote stress redistribution and further energy dissipation during crack growth. This deflection-based mechanism works synergistically with hysteresis driven dissipation to prevent catastrophic failure and preserve mechanical integrity under applied stress [90].

### Double network and interpenetrating network architectures

A double network (DN) hydrogel is built up of two interpenetrating polymer networks (IPNs) which are intentionally designed to serve distinct mechanical functions: a solid and brittle first network and a soft and expansive second network [91]. The brittle network undergoes fracture when it is subjected to mechanical loading, and therefore, it absorbs the energy through ruptures of sacrificial bonds more curiously, whereas the soft ductile network remains stable and resilient. This brittle-ductile synergy allows for deformation-induced toughening which occurs through redistribution of stress and inhibition of catastrophic crack propagation [92].

In comparison, an IPN is composed of two or more polymer networks, physically interlaced but generally has no predefined hierarchy of sacrificing load bearing elements [93]. Consequently, IPNs do not intrinsically display the same level of controlled energy dissipation as DN hydrogels unless other means of toughening are added [94]. Mechanistically, DN hydrogels are intentionally designed to maximize energy dissipation by network asymmetry and optimized cross-link density, whereas IPNs mainly utilize network interlocking and cooperative deformation for mechanical reinforcement [95]. Building on this network hierarchy, DN hydrogels dissipate mechanical energy by mainly sacrificial bond rupture by allowing the brittle first network to fracture preferentially under mechanical loading. The breaking of these sacrificial bonds soaks up a great amount of applied mechanical energy, and thus, reduces the concentration of stress and delays catastrophic failure. This dissipation of energy is especially efficient when sacrificial energy is released by amorphous inorganic or weakly bonded domains to a much greater extent than the elastic polymer network upon deformation [96].

The crack tip process zone in DN hydrogels is described by a two-stage energy dissipation process, in the pre-yield zone local necking separates both fractured and unfractured material, and in yield regime the whole necking process results in a macroscopic fracture [97]. The dynamic properties of polymer chains, especially solvent viscosity and chain relaxation behavior, impact stress redistribution and energy dissipation in the necking zone, hence, affecting its development and overall fracture toughness of the hydrogel [98]. The use of mechanochemical methods allows visualizing the cutting of bonds in the damage zone and creates the spatial maps of stress and strain, as well as energy elution that clearly show the spatial variations close to the crack tip [99]. The intrinsic fracture energy is influenced by loading history as prestretching changes the role of brittle network in total fracture energy [100].

The brittle-ductile network synergy further increases the toughness by coupling a rigid and highly cross-linked primary network with a pliant and extensible secondary network [101]. Conversely, upon deformation, the brittle network dissipates the energy in a controlled way via fracture, whereas the ductile network allows load bearing and large strains for preserving the macroscopic integrity [102]. Incorporation of dynamic noncovalent interactions brings in the reversible sacrificial links which allow the repeated cycles of bond rupture and reformation that lead to persistent energy dissipation and enhanced tolerance of damage [103]. Together, sacrificial bond rupture and brittle-ductile synergy enable DN hydrogels to dissipate energy efficiently on multiple length scales to provide a material with exceptional toughness and fracture resistance [104].

Cross-link density, and the relative stoichiometry of the constituent networks are the most important factors that control the toughness and fracture resistance of double-network hydrogels. An increase in the percentage of cross-links in the brittle primary network increases stiffness and reduces extensibility, which may inhibit plastic deformation and energy loss in case the material has too many cross-links. Conversely, decreasing cross-link density increases the mobility of the chains, and allows for sacrificial bond rupture and more energy dissipation during deformation [105].

The network ratio is an equally important factor, as the first, rigid network largely dissipates energy by controlled fracture, whereas the second, ductile network preserves the elasticity and load-bearing capacity. Increasing the proportion of the second network results in higher stretchability and a higher fracture resistance by permitting a more efficient redistribution of stress after damage to the first network [101]. Fine-tuning this ratio allows optimizing fracture energy and toughness without any loss in structural integrity [106]. Further physical entanglements or supramolecular interactions contribute to the reinforcement of toughness by the introduction of reversible dissipation of energy and rapid mechanical recovery [107]. Advanced architectures including fibrillar connected DN hydrogels have shown that controlled cross-linking and network composition can be used to attain high strength and toughness with minimal hysteresis [108].

### Hybrid crosslinking strategies

Covalent crosslinks provide permanent chemical connections that impart hydrogel assemblies with high strength and rigidity by strengthening their structural frameworks [109]. However, due to their innate stiffness and irreversibility, covalent bonds have a limited ability to dissipate energy during deformation and can cause brittle rupture when used alone. To negate this limitation, sacrificial conformations are commonly designed into covalent networks to dissipate partial energy without reducing the integrity of the overall structure of the network [110]. Conversely, ionic crosslinks have the dynamic property with reversibility that allows them to dismantle and rearranging upon mechanical loading, and therefore, the ability to produce effective energy dissipation and tough reinforcement. In the ionically crosslinked hydrogels, reversible electrostatic interactions harness mechanical energy, while supporting self-recovery and damage tolerance [111]. Ionic interactions also trigger phase separation and viscoelastic damping of polyelectrolyte hydrogels, and by doing this, more energy is dissipated during the deformation process [112].

Combining both crosslink types, hybrid ionic-covalent networks combine the strong behavior of covalent bonding with the dissipation behavior of ionic interactions resulting in hydrogels with increased toughness, fatigue resistance and mechanical stability [113]. Mechanistically, fatigue resistance and self-recovery in hybrid ionic-covalent hydrogel are due to the dynamic rupture and formation of ionic bonds during cyclic loading that act as sacrificial sites of energy-dissipation but leave the integrity of the covalent network intact [114]. During repeated deformations, ionic bonds are selectively broken to take up mechanical energy and prevent stress accumulation and reform during unloading to allow effective structural recovery without irreversible damage [115, 116]. Stress redistribution and viscoelastic damping are further promoted by cooperative physical interactions such as hydrogen bonding, ionic clustering, and metal and ligand coordination, which increase resistance to fatigue induced failure [117, 118]. This reversible reconstitution of molecular interactions also allows hybrid networks to perform mechanical activity over prolonged cyclic loading, unlike purely covalent systems which accumulate irreversible damage [111], and provides an effective balance between structural robustness and energy dissipation, making them particularly attractive to biomedical applications requiring mechanical loads [113].

### Hydrogel design summary for regenerative medicine

Multi-mechanism toughening approaches are desirable since none of the mechanisms can independently provide high strength, toughness, fatigue resistance and damage tolerance across hydrogel network [119, 120]. The hybrid designs combine complementary energy dissipation mechanisms, which include sacrificial bond rupture, hierarchical network structures and crack-deflection mechanisms, thus, allowing mechanical energy to be taken up on a variety of length scales [121, 122]. This synergistic dissipation averts localized failure and minimizes the brittleness or instability commonly observed in single-mechanism hydrogels [108]. Multi-mechanism strategies, therefore, offer a better and more certain route to the achievement of mechanically robust and durable hydrogels that can be used in the biomedical needs [85].

In PNIPAM-based systems these principles have been converted into practical material designs. For example, DN PNIPAM hydrogels containing a stiff base network and a soft secondary network have shown significantly improved mechanical strength and fracture tolerance through a stress redistribution mechanism and sacrificial network rupture mechanisms [20]. Likewise, covalent and noncovalent systems, such as ionic coordination or hydrogen bonding, known as PNIPAM-based double-crosslinked systems, exhibit enhanced tensile and compressive performance while retaining flexibility under cycled deformation [23]. Dynamic bonding strategies have also been used, in which reversible ionic or hydrogen-bond interactions are introduced into PNIPAM networks to simultaneously increase the toughness and self-healing efficiency, and thus, damage tolerance under repeating loading [123]. Moreover, nanocomposite PNIPAM hydrogels with graphene oxide (GO) or other inorganic fillers exhibit enhanced mechanical robustness and fatigue resistance by crack deflection and interfacial stress transfer mechanisms [124]. Collectively, these examples show how the ideas for toughening are directly applied in the design of PNIPAM hydrogels to meet the mechanical needs for dynamic wound environments.

Table 2 summarizes the mechanical performance and design strategies of PNIPAM-based hydrogel dressings.

**Table 2:** Mechanical performance and structural design strategies of PNIPAM-based hydrogel dressings.

Mechanical aspect	Reported performance/Feature	Structural or material strategy	Reference
LCST behavior	32°C -Thermoresponsive volume change	Core property of PNIPAM	[48]
Elastic modulus	Increases with crosslinking and polymer content	MXene, clay, thiol-ene, covalent bonding	[125–127]
Swelling behavior	High hygroscopic efficiency (up to 80%)	PNIPAM/PPy formulation	[63]
Durability (wet/dry)	Humidity dependent softening/stiffening	PEG foams, xanthan-CS hybrid network	[65, 66]
Mechanical toughness	Enhanced fracture resistance	GO nanocomposite reinforcement	[128]
Thermal stability	Improved thermo-mechanical integrity	Silica nanoparticle incorporation	[129]
DN architecture	High strength via two-network synergy	Rigid first network + elastic second network	[130]
Self-healing capability	Reversible mechanical recovery	Dynamic covalent bonds (Schiff base, disulfide)	[9, 131]
Electrical/mechanical coupling	Increase in conductivity and toughness	CNT and GO integration	[132,133]
Injectability	Maintains mechanical integrity after injection	PNIPAM + CS liquid bandage systems	[134]

The elastic modulus of PNIPAM gel dressings forms a decisive factor in their mechanical characteristics, especially in the medical and wound healing circumstances. PNIPAM hydrogels undergo temperature triggered mechanical changes, which makes them applicable not only in wound dressing technologies but also in sensor applications [36, 47]. Simulation studies have shown that increased crosslinking and polymerization can expand the elastic modulus, strengthening PNIPAM hydrogels [126, 135].

## Adhesion mechanisms of PNIPAM-based hydrogels for regenerative medicine

### Interfacial adhesion mechanisms

In wound dressing procedures, hydrogel-based dressings with ideal adhesive characteristics should have several defining features, such as sufficient bonding, injectability, self-healing behavior, fault tolerance, repeat tissue adhesion and time dependent adhesive properties.

The adhesive behavior of PNIPAM-based hydrogels on biological tissues is largely determined by interfacial physical and chemical interactions between the hydrogel network and the substrate. The composition of polymer has a central effect because the functional group that can engage in hydrogen bonding, electrostatic interaction and bridging between the particles with tissue components positively increases the interfacial adhesion [136]. As for polymers, like chitosan and gelatin, containing abundant polar or charged groups, they usually exhibit an increased interaction with the tissue surfaces [18, 137].

The density of crosslinking also has effects on adhesion as it controls the network integrity and surface compliance. Hydrogels with appropriate crosslinking density provide enough mechanical stability and maintain close contact at the interface, which is especially significant in wet environments [18]. In PNIPAM-based systems, the choice of polymers and crosslinkers, therefore, determines the balance between interfacial bonding and tensile strength, and thus, has a direct effect on tissue adhesion behavior.

### Network structure and thermoresponsive effects

PNIPAM networks have been studied to deswell quickly with network contraction and water content reduction at temperatures above the LCST [138]. This increase in density of structure caused by thermally induced modification of mechanical condition of the hydrogel creates moderate rigidity, but leave it sufficiently flexible to adapt conformally to interfaces with tissue surfaces.

The relationship between the network contraction and maintenance of compliance increases the interfacial contact and load transfer between the hydrogel and tissue interface, which promotes efficient adhesion when exposed to physiological conditions [139]. In addition, the transition toward LCST is reversible allowing PNIPAM hydrogels to tune swelling behaviors, mechanical integrity and adhesion in response to changes in temperature near body temperature [140].

### Adhesion of hydrogels in dry and wet environments

In dry conditions, the noncovalent interfacial interactions and internal network cohesion are the main controls of hydrogel adhesion to tissues. The forces that drive the adhesion include hydrogen bonding, van der Waals, polymer entanglement and π-π interactions, and one of the predominant interactions usually determines the overall adhesive strength [141, 142]. The network of hydrogel should have cohesive strength that can withstand mechanical stress and prevent detachment of the hydrogel during tissue movement [143]. Other processes, such as topological entanglement of sol to gel transition, adsorption of macromolecules through solid particles, also play an additional role in strong adhesion in dry environments [136, 144].

Conversely, during wet physiological conditions swelling and hydration are very critical factors in defining adhesion performance. In most cases, swelling reduces the strength of interface bonds, thus, raising the chances of stress failure of adhesion in the presence of body fluids or blood [145]. To maintain adhesion in wet conditions it is critical to reduce swelling as well as contain hydration. Some of the strategies reported in the literature include limiting water intrusion, increasing energy dissipation in the network and absorbing interfacial water that helps to promote increased adhesion in hydrated conditions [139, 145, 146]. Based on this, the unique adhesion properties of hydrogels in dry and wet tissues can be controlled by the interactions between swelling control, hydration management and network architecture.

Hydrogel-based devices are required to be flexible, strong and elastic in dynamic environments to ensure adhesion. Tough and flexible hydrogels tend to be better wound dressing agents. The adhesion potency of PNIPAM-based hydrogels depends on the presence of biological fluids, ionic strength, as well as pH. The best hydrogels in practice are those that can change with a changing environment and still maintain adhesion [147].

### PNIPAM-based adhesive systems for regenerative medicine

Representative PNIPAM-based hydrogel systems illustrate the potential to leverage thermoresponsive network design and interfacial chemistry to provide tunable tissue adhesion. Yu et al. prepared a PNIPAM/Sodium Alginate/GO hydrogel dressing where the volumetric shrinkage of the hydrogel and its adhesiveness was regulated by near-infrared irradiation. The addition of bridging polymers, including chitosan, resulted in high tissue adhesion strength of 7.86 ± 1.22 kPa and allowed adhesion in a region-specific manner. The resulting double crosslinked matrix facilitated sutureless wound healing *in vivo*, thus, suggesting the possibilities of contractile PNIPAM hydrogels to regulate tissue adhesion [23].

**Figure 3:**
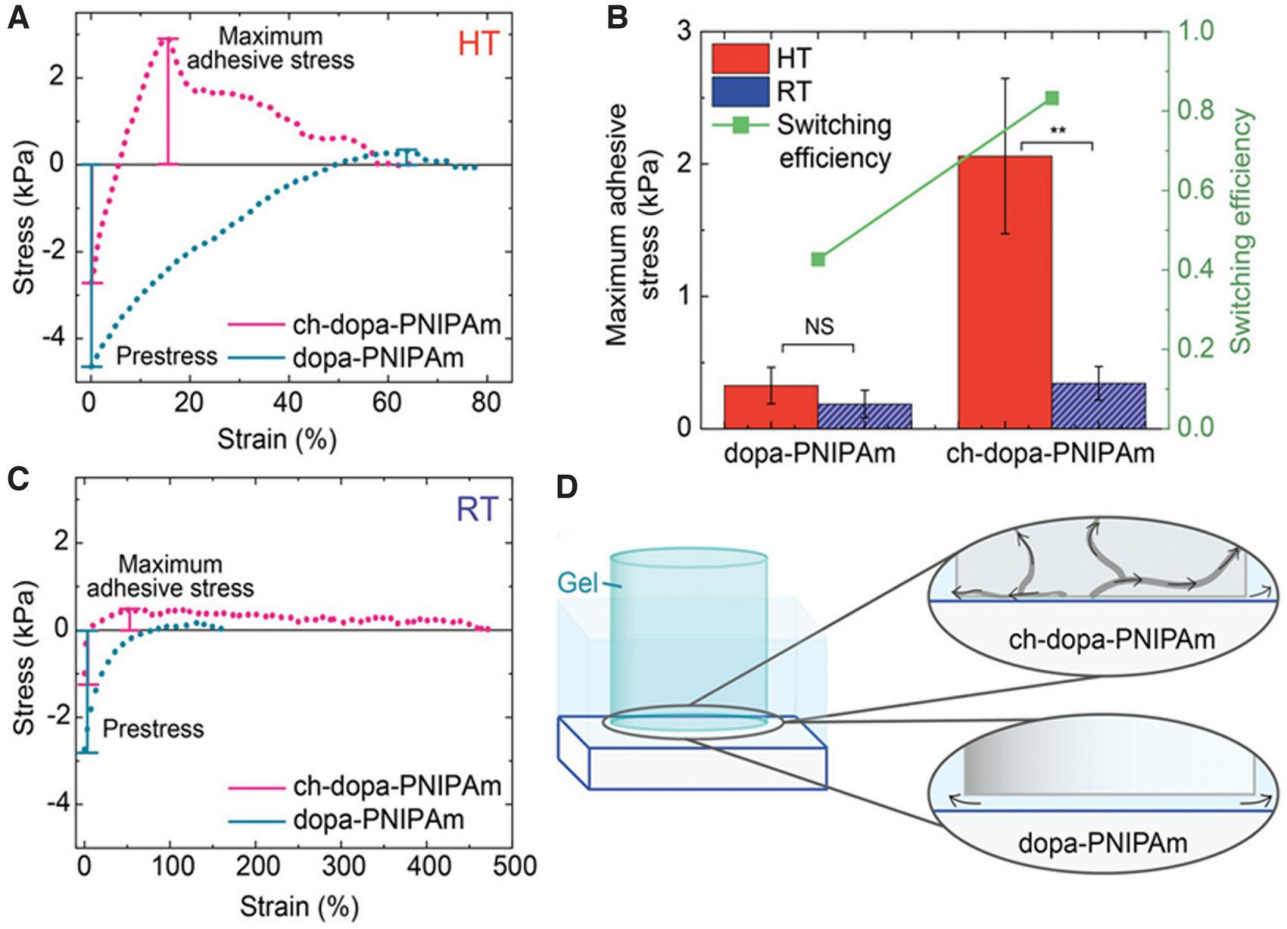
High switching efficiency underwater adhesion in a channeled hydrogel. HT: high temperature (45°C) RT: Room temperature. (**A**) HT and (**B**) RT adhesive stress as a function of strain for ch-dopa-PNIPAM and dopa-PNIPAM on a glass substrate, with prestress and maximum adhesive stress marked. Hydrogel composition: 10 wt.% NIPAM, 1 mol.% DPMA and 0.1 mol.% BIS relative to NIPAM. Agarose (0.5 wt.%) was used to prepare ch-dopa-PNIPAM. (**C**) Average maximum adhesive stress of five measurements of dopa-PNIPAM and ch-dopa-PNIPAM at RT and HT, as well as the switching efficiency. (**D**) Illustration of improved water removal from interface due to the channeled structure. Reproduced with permission [45], Copyright 2023, *Advanced Functional Materials*.

Similarly, a channeled dopamine-modified PNIPAM hydrogel (ch-dopa-PNIPAM) was designed to achieve temperature-controlled switchable underwater adhesion through phase transitions. The combination of bio-inspired dopamine moieties and microchannel structures enabled an efficient water transport at the interface and obtained increased adhesion strength and switching efficiency as illustrated in Figure 3. The highest adhesive stress of 6.2 kPa and switching efficiency of 0.91 were reached in this system, which were better than the traditional dopamine-containing PNIPAM hydrogels [45]. Collectively, these examples demonstrate that structural design and thermoresponsive behavior jointly govern adhesion performance in PNIPAM‑based systems.

Although the above examples have shown that the thermoresponsive network design and interfacial chemistry determine the adhesion performance, the mechanical response to PNIPAM-based hydrogels is vital in dictating the strength and failure behavior of adhesion. Since adhesion durability is affected by viscoelastic deformation and energy dissipation, the following section briefly describes several rheology indicators, which are relevant to PNIPAM wound dressings.

In Table 3, a comparative overview of representative PNIPAM-based hydrogel dressings, including their adhesion properties (dry/wet), switching properties and interfacial performance is provided.

**Table 3:** Adhesive and interfacial performance of PNIPAM-based hydrogel dressings.

Adhesion aspect	Measured/Observed performance	Enhancing strategy	Reference
Adhesive strength (wet tissue)	7.86 ± 1.22 kPa	Double crosslinked PNIPAM-CS hydrogel	[23]
Switchable adhesion	Max adhesive stress of 6.2 kPa	Channeling via PNIPAM and dopamine comonomers	[45]
Dry/wet adaptability	Stable adhesion across hydration states	Composite and nanocomposite designs	[136, 139, 144–147]
Injectable adhesive behavior	Hemostatic, antibacterial wet adhesion	PNIPAM-based liquid bandages	[134]

## Rheological and viscoelastic properties of PNIPAM hydrogels for regenerative medicine

Rheology of PNIPAM-based hydrogels is a decisive factor affecting its adhesion properties and viability in wound dressing. In soft hydrogel systems, viscoelastic properties determine the integrity of the network, deformation response and energy dissipation of the network during the interfacial contact and detachment processes. Rheological analysis, therefore, provides mechanistic understanding of adhesion stability and breakdown whilst providing a practical paradigm of assessing injectability and handling under physiological environments.

### Viscoelastic behavior relevant to adhesion

Viscoelastic properties of hydrogel measured by the storage modulus (*G*′) and loss modulus (*G*″) play a vital role in controlling tissue adhesion strength and failure behavior. *G*′ represents the solid or elastic property of the hydrogel, and *G*″ is the viscous characteristic of the hydrogel. Increased *G*′ is typically linked to the rise in stiffness and mechanical strength, which, in turn, can increase tissue adhesion by raising mechanical stability [148]. Experimental results have proved that optimization of the viscoelastic parameters in the hydrogels leads to better tissue adhesive properties. As an example, it is desirable to observe that systems with large storage moduli along with balanced viscoelastic behavior have higher mechanical endurance and adhesion properties in the biomedical applications [148, 149]. Moreover, hydrogels that are especially prepared to maintain desirable viscoelastic behavior in wet conditions, e.g. swelling resistant formulations exhibit strong adhesion stability in body fluids [145].

Rheological studies such as strain (amplitude) and frequency sweeps are commonly used to understand the viscoelastic properties of hydrogels by measuring *G*′ and *G*″ in real-time as the degree of deformation of the gel is increased. Strain sweep experiments are used to identify the linear viscoelastic region (LVER), which is defined as a strain range over which the hydrogel network maintains the structural integrity and *G*′ and *G*″ do not change with respect to strain amplitude. The LVER subsequently determines the appropriate strain conditions used for successive frequency sweep studies aimed at the determination of the time-dependent viscoelastic behavior of the gel [150]. All these data indicate the paramount importance of realizing the reasonable balance of elastic and viscous responses to maintain the adhesion strength and nip the threat of adhesive failure in tissue adhesive hydrogels [145, 148, 149].

### Shear response and injectability of hydrogel dressings

The shear-thinning effect is an important determinant of injectability of hydrogel wound dressing because it allows a significant viscosity reduction when subjected to shear force, enabling easy extrusion of the sample by needles or catheters during minimally invasive procedures [151]. This rheological property facilitates the passage of fluid when injecting it, but at the same time it reduces resistance and ensures that the delivery device is not clogged. Reproductive reassembly of hydrogel network following injection is critical to restore the functionality and re-establishing mechanical integrity at the target site. This type of recovery is obtained through reversible interactions and dynamic cross-linking processes that allows the hydrogel to reform of its initial structure when the shear forces are removed [152–154].

**Figure 4:**
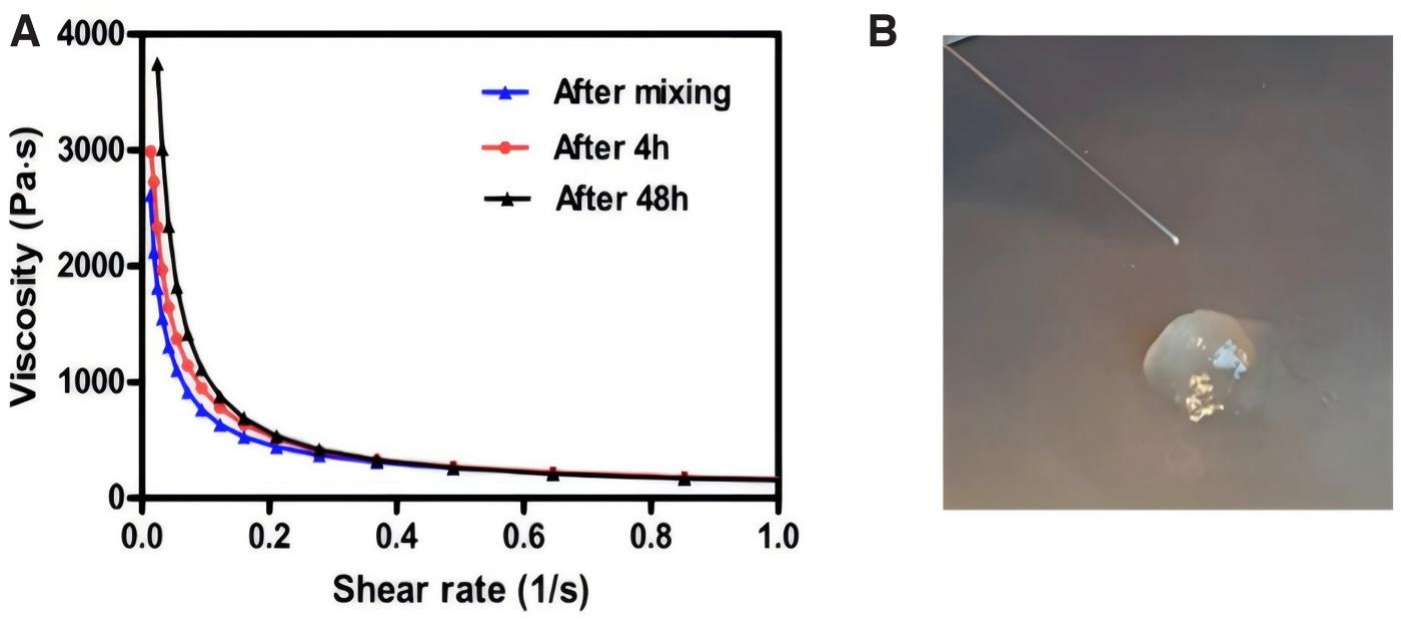
Rheological and injectability behavior of an injectable collagen hydrogel. (**A**) Viscosity as a function of shear rate, demonstrating pronounced shear-thinning behavior at different time points after gel preparation. (**B**) Photograph showing successful extrusion of the hydrogel through a 27 G needle. Reproduced with permission [155], Copyright 2024, *ACS Applied Materials & Interfaces*.

The balance between shear-thinning during injection and rapid post-injection structural recovery makes it possible to deliver hydrogels efficiently while forming a stable depot *in situ* [151]. These 2-fold features, including flowability and rapid recovery, is thus, critical to the effective application of injectable hydrogel wound dressing as it forms the basis of both handling behavior and long-term functional stability after placement [155]. Macroscopic injectability using very fine needles and shear-thinning behavior enabling smooth injection has been clearly demonstrated in injectable collagen-based hydrogels (Figure 4), thus, exemplifying the general rheological principles that are relevant to the design of injectable hydrogel wound dressings.

### Role of viscoelastic dissipation in adhesion failure

Viscoelastic energy dissipation plays a central role in dictating how adhesive failure occurs in hydrogels, that failure occurs either cohesively in the bulk or at a hydrogel-substrate interface. Mechanical energy dissipation is an effective process in deformation that allows hydrogels to survive the mechanical stresses without internal fracture, therefore, preferring the cohesive to interfacial failures [156]. This dissipation is the result of the reversible molecular interactions and structural configurations to enable absorption of stress during loading and unloading cycles.

Adhesive performance is also balanced by the interaction between viscoelastic dissipation and damage accumulation. Viscoelastic dissipation dominates at lower stress levels or shorter loading times, leading to stable adhesion and cohesive failure, whereas damage dissipation increases with an increase in stress levels or prolonged loading, increasing the likelihood of interfacial failure [157]. Therefore, to control adhesion durability and failure modes in adhesive systems composed of hydrogel it is necessary to design materials that can be used to control viscoelastic dissipation [156, 157].

## Approaches to improve PNIPAM-based dressings for regenerative medicine

PNIPAM hydrogels offer unique thermoresponsive handling for wound care, but unmodified networks often lack the mechanical robustness and interfacial reliability required for real wounds, especially under hydration, repeated deformation and motion at joints. Therefore, most advanced PNIPAM dressings rely on engineering trade-offs: increasing strength and toughness without sacrificing conformability, injectability or temperature-triggered detachment. In this section, reinforcement and adhesion strategies are synthesized comparatively, focusing on (i) what each approach improves most, (ii) where it underperforms (e.g. wet durability, long-term stability, biocompatibility) and (iii) which wound scenarios it best fit (dry vs exudative wounds, static vs highly mobile sites, patch vs injectable delivery).

### Approaches to improve mechanical properties

Mechanical reinforcement of PNIPAM-based hydrogels is commonly achieved through four design routes: (i) incorporation of hybrid nanocomposites, (ii) designing DN hydrogels, (iii) dynamic crosslinking and (iv) incorporation of conductive materials. Each route improves specific mechanical metrics (e.g. stiffness, toughness, fatigue resistance or functional stability) but introduces trade-offs in thermoresponsive behavior, deformability, processing complexity and translational safety.

**Figure 5:**
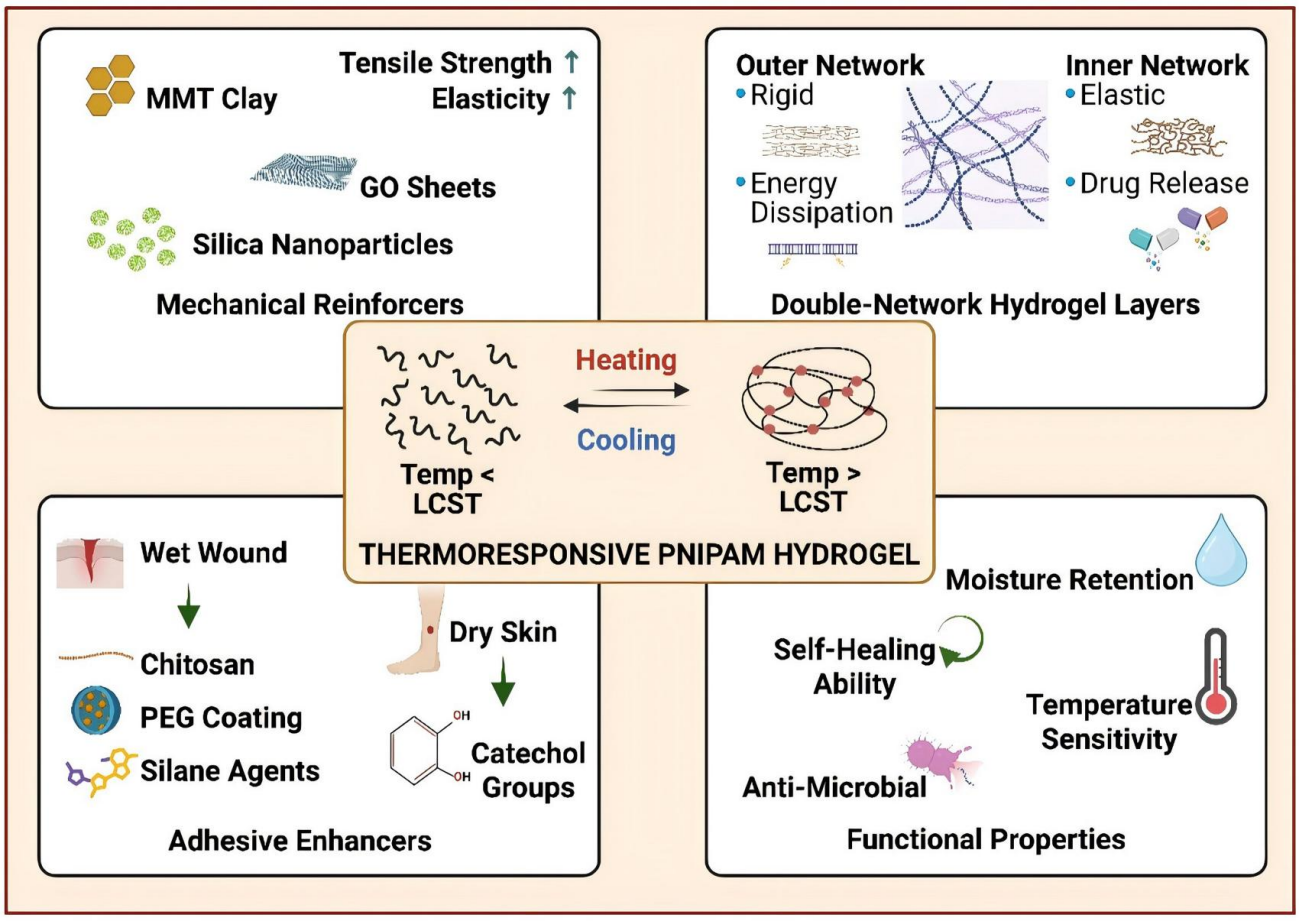
Schematic illustration of multifunctional PNIPAM-based hydrogel dressing showing thermo-responsiveness, mechanical reinforcers, adhesive enhancers and adaptation to dry and wet tissue environments.

As illustrated in Figure 5, PNIPAM-based hydrogel dressings leverage thermo-responsive behavior, nanocomposite reinforcement and surface modifications to achieve enhanced mechanical performance and tissue adhesion across both dry and wet wound environments.

For PNIPAM-based hydrogel wound dressings, several specific strategies can be employed to enhance their mechanical properties:

#### Incorporation of hybrid nanocomposites

**Figure 6:**
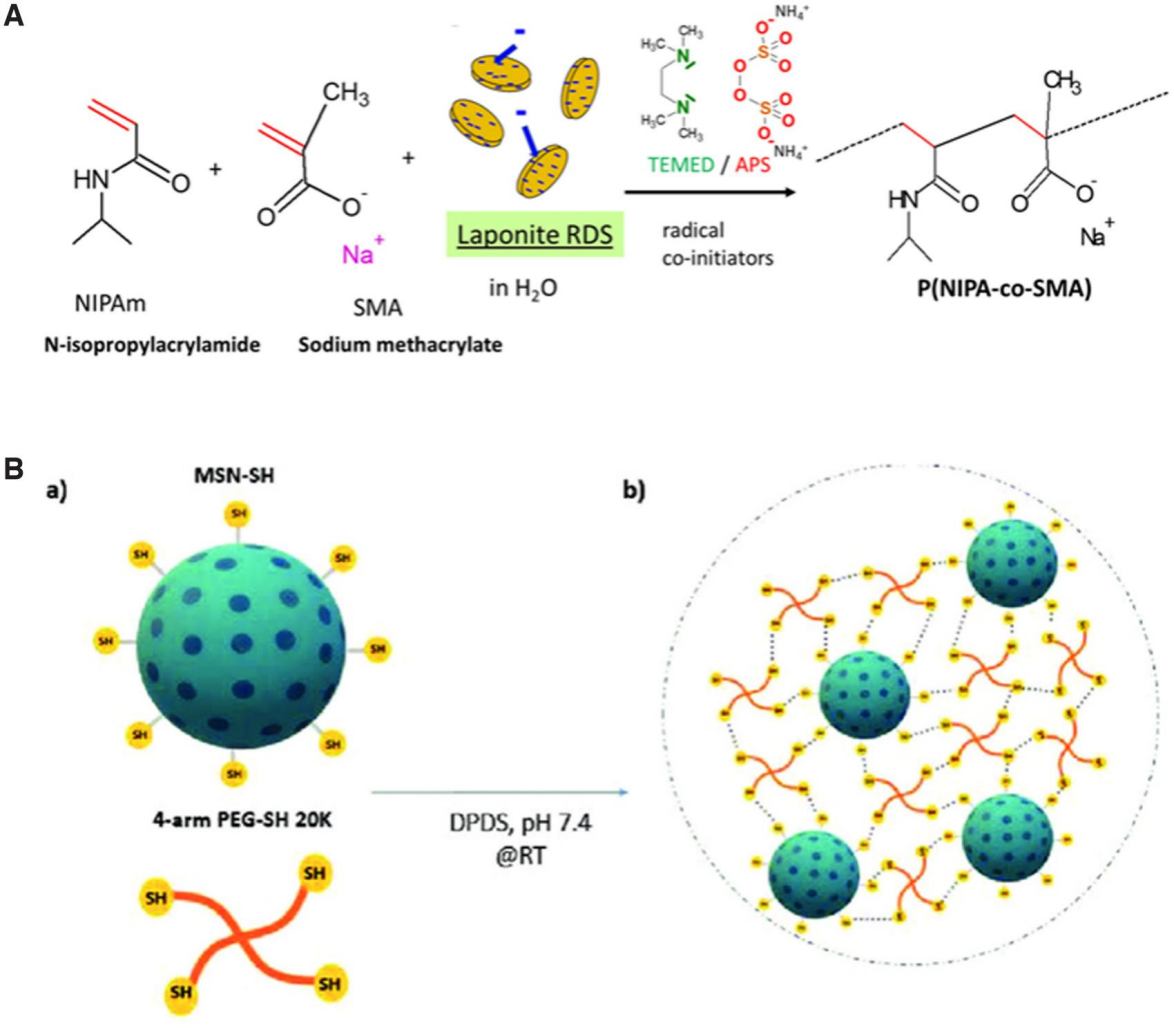
(**A**) Synthesis of the nanocomposite PNIPAM/clay hydrogels sensitive to temperature and pH. (**B**) Formation of MSN-PEG nanocomposite hydrogels via dynamic covalent interactions. (a) Chemical structure of MSN-SH and 4-arm-PEG-SH. (b) Hydrogel formation through the thiol-disulfide exchange reactions at room temperature. Reproduced with permission [159], Copyright 2021, Elsevier. [160], Copyright 2021, Nanoscale.

Hybrid nanocomposites greatly boost the mechanical strength of PNIPAM-based hydrogels mainly because of the inclusion of other materials that increase surface interactions and wettability [158]. Incorporation of nanoparticles e.g. clay nanoplatelets, carbon nanotubes (CNTs) as well as other nanostructures can enhance cross linking opportunities in the hydrogel matrix, thus, enhancing elasticity and modulus. As an example, nanoplatelets of clay that have been physically cross-linked to PNIPAM give high tensile and mechanical strength and can be stimulated to respond to two stimuli simultaneously, temperature and pH, which is necessary when used in soft actuators or drug-delivery systems (Figure 6) [159]. The hydrogel also has nanocomposites that add some more crosslinking sites to the network to improve its tensile strength, elasticity and toughness. Having hydroxyl and carboxyl functional groups, GO is strongly bonded to the hydrogel matrix by hydrogen bonding. Such reversible bonds go into and out of solution constantly, making the hydrogel hold up its own structure in even varying mechanical stress, as well as enhancing its anti-deformation ability [128]. Composite hydrogels with incorporated graphene-based porous networks with PNIPAM demonstrate greater multifunctionality; the conductive and mechanical properties of graphene are coupled with the stimuli-responsive properties of PNIPAM to produce robust, elastic and responsive composite hydrogel [161].

The advantages of the use of silica nanoparticles in PNIPAM hydrogels are not recent. Silica nanoparticles can be used as a successful cross-linker, thus, increasing the storage modulus and total mechanical strength of the material. As an example, mesoporous silica nanoparticles (MSNs) have been described to enhance the storage modulus of hydrogels, in the case of dynamic crosslinkers, with an improvement of 1.3 ± 0.3 kPa to 32 ± 5 kPa in terms of the storage modulus (Figure 6) [160]. Combining crosslinking agents with clay and MXene substances can enhance the hydrogel’s tensile strength and elongation, thereby improving its elasticity and adhesive capabilities [125]. Incorporating MXene into PNIPAM hydrogels increases their thermal conductivity and responsiveness, which can enhance sensor integration in advanced wound dressing applications [162].

Additionally, the covalent bonding of amine functionalized silica nanoparticles within DN hydrogels has been reported to increase compressive strength from 0.10 MPa to 1.28 MPa, indicating a significant improvement in mechanical resilience [163]. Furthermore, the grafting of polymer chains onto silica nanoparticles can enhance particle dispersion within the hydrogel matrix, further improving mechanical properties [164]. The incorporation of more biodegradable and biocompatible polymers, especially the hydroxypropyl methylcellulose (HPMC) also expand the mechanical properties and tunable swelling attributes of the hydrogel construct further [52].

#### Designing DN hydrogels

DN construction is a popular and effective approach to the field that can be used to enhance the mechanical properties of hydrogels like PNIPAM-based formulations that, by nature, have low toughness and mechanical strength in comparison to most other hydrogels. The initial network, in DN hydrogels, is extensively crosslinked, and the second should be loosely crosslinked. The effect of this is substantial increase in resistance to tearing and rupture making DN hydrogel wound dressing suitable where durability is paramount [130].

In a dry and wet environment, DN hydrogels show high bond adhesion. The mechanical skeleton is provided by the first network and the adaptability to wound surface protrusion is ensured by the second network that ensures an uninterrupted adherence of the hydrogel to the surface of the wound, without which wound healing does not take place. Thus, the hydrogel dressing also provide good adhesion under the dynamic environment due to the combination of these two mechanisms [165]. The network composition and crosslinking density of any network forming a DN hydrogel can be controlled to exact specifications to suit a particular application need [135].

**Figure 7:**
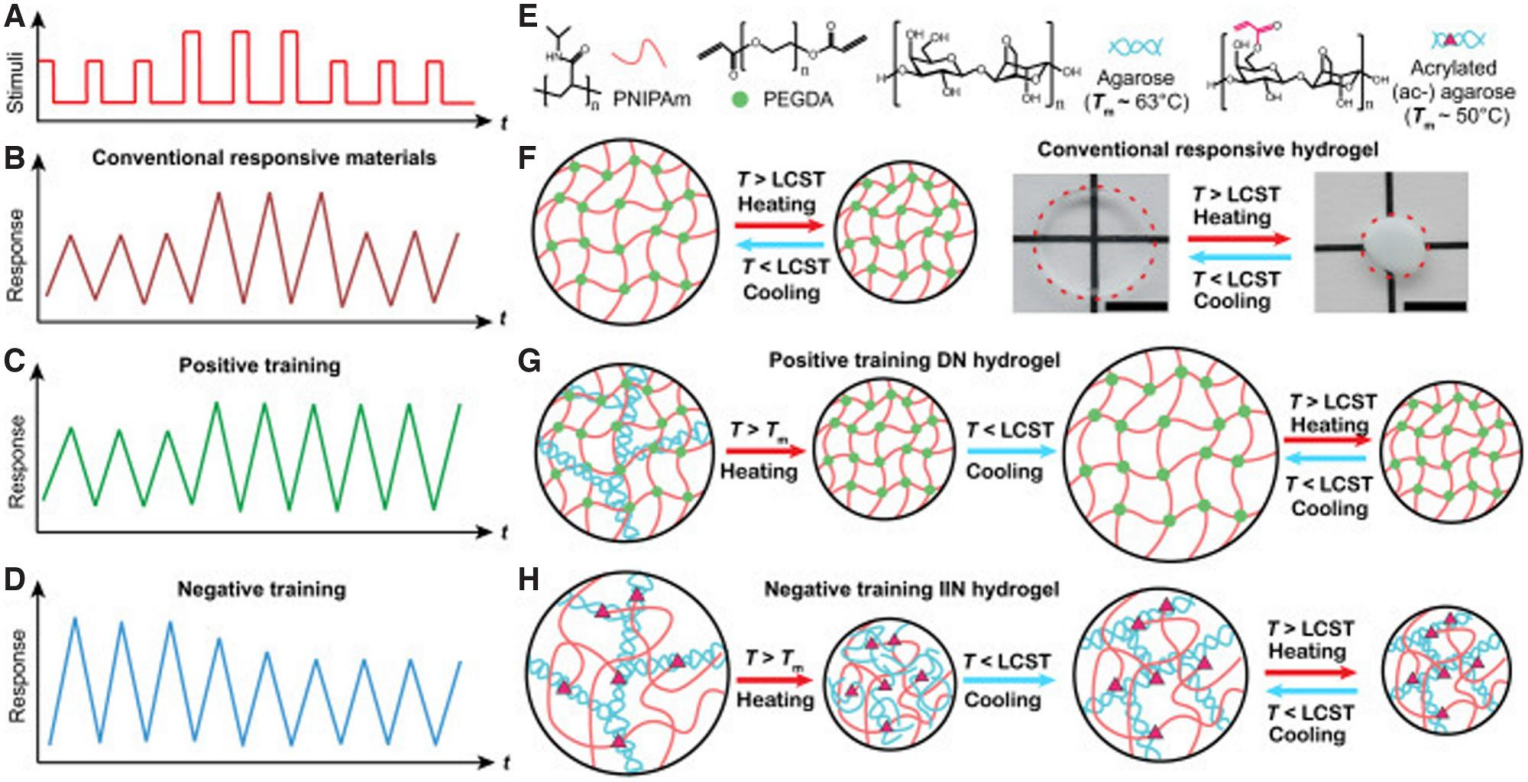
(**A**) Stimuli of varying intensities. Responses of (**B**) conventional responsive materials, (**C**) positive training material and (**D**) negative training material induced by the stimuli in (A). (**E**) Compositions of the trainable hydrogels. (**F**) A single network PNIPAM gel showing conventional thermoreversible volume changes corresponding to (B). scale bar: 10 mm (black) for the photograph. (**G**) A DN agarose/PNIPAM hydrogel showing positive training corresponding to (C). (**H**) An interconnected interpenetrating network (IIN) ac-agarose/PNIPAM hydrogel showing negative training corresponding to (D). Reproduced with permission [166], Copyright 2023, *Nature Communications*.

Figure 7 shows a PNIPAM-agarose dual-network hydrogel whereby agarose is gelled initially, and then, radical polymerization of PNIPAM is carried out in the presence of a cross-linker, thus, giving rise to a chemically cross-linked PNIPAM network that is physically cross-linked by a network of agarose. The DN exhibits a volume phase transition around 35°C, but the volume change is initially restricted by the agarose network. When subjected to a thermal procedure, known as thermal training, namely heating to a temperature necessary to melt agarose (which is of significance in the procedure), the agarose network is removed or relaxed, allowing the PNIPAM network to expand and shrink to a much larger extent, which is accompanied by changes in mechanical. This DN design demonstrates how network hierarchy and thermal history can be used to tune thermoresponsive mechanics for adaptive soft materials [166].

Han *et al*. took the fabrication of densely crosslinked PNIPAM/keratin DN gels using a mixture of covalent and ionic crosslinking strategy. The gels have a strong fluid uptake, swelling ratios ranging between 2600% and 4600% with an increase in keratin content and they have thermoresponsive behavior that facilitates a self-stripping dressing idea. Moreover, the matrices can be post loaded with chlorhexidine acetate, which enables multiresponsive drug delivery by controlling release based on temperature, pH and reactive oxygen species and superior tissue regeneration compared to a commercially available film in a rat skin defect model [167]. In another study, Li et al. prepared strong and thermosensitive PNIPAM-based DN hydrogel which contains a highly rigid poly (2-acrylamido-2-methylpropanesulfonic acid sodium salt) PNaAMPS first network and a highly flexible P(NIPAMco-AAm) second network. The resulting materials have a mechanical strength of 0.83–1.37 MPa, have a time changing swelling, and can be biocompatible up to the physiological temperatures. Crystal violet loading gives it bactericidal properties against *Escherichia coli* (*E. coli)*, and thus, demonstrates how dual network architecture can be used to both strengthen mechanical toughness and provide antimicrobial properties [135].

However, the process of forming these networks can be complex and time-consuming, and controlling the mechanical properties remains challenging [105]. Recent research has also explored the use of unconventional monomers and dynamic covalent bonds within DN hydrogels. This direction has given rise to hydrogels that are self-healing, stretchable and tough in some way, that can endure harsh conditions and provide new opportunities in how multifunctional hydrogels can be created.

#### Dynamic crosslinking

Reversible crosslinking of PNIPAM-based hydrogels has numerous applications in biomedical engineering, enhancing their functionality for both therapeutic and structural purposes. The creation of self-repairing hydrogels that can mechanically support tissue engineering, automatically repair injury and adjust to physiological conditions is made possible by this technology. In regenerative medicine applications, neural tissue repair and self-healing hydrogels are especially useful [168]. Injectable hydrogels, like thiolated alginate hydrogels, which can encapsulate cells and be used in 3D bioprinting, offer precise control for tissue engineering applications [169]. Strong adhesive and self-healing qualities are demonstrated by bioinspired hydrogels with dynamic crosslinking, which enable sutureless wound closure and accelerate the healing process [9].

Hydrogel frameworks are commonly complexed with dynamic covalent bonding (e.g. Schiff base, boronate, disulfide, etc.). These reversible covalent interactions yield self-repair properties, and the hydrogel can reform its network structure once it is damaged. Upon cracking or tearing of the material, the dynamic bonds are broken and then re-formed, thus, regaining hydrogel architecture at a cost of temporary mechanical strength. The property is especially beneficial in the wound dressing case because it enables the material to continue to work even when exposed to long-time mechanical stresses or physiological stresses or strains [131].

Exquisite control of the mechanical characteristics of hydrogels is possible via controlled dynamic covalent chemistry, making the material sensitive to environmental impacts. An example is reversible ionic bonding of hydrogels, which allows the tight control of stiffness based on changes in either pH or ionic concentration, a goal that is of particular importance in wound bed conditions when moisture levels and acidity can change [170]. This comes with enhanced flexibility to improve the functionality of hydrogel in terms of adhesion and stability in wet and dry environments. Besides, addition of covalent bondages through thiol-ene reactions significantly enhances the stiffness and mechanical strength of the gel, thus, it is possible to manipulate the material to fit different tissues with their respective compliance needs [127]. Water and ionic liquid solvent systems are also less polarizable, and thus, enhance noncovalent interactions and enhance mechanical properties and self-healing ability [171]. The possibility to form temperature-controlled viscoelastic responses of PNIPAM hydrogels due to a ratio of monomers to the crosslinker demonstrates the applicability of these polymers across the range of biomedical implications [172].

**Figure 8:**
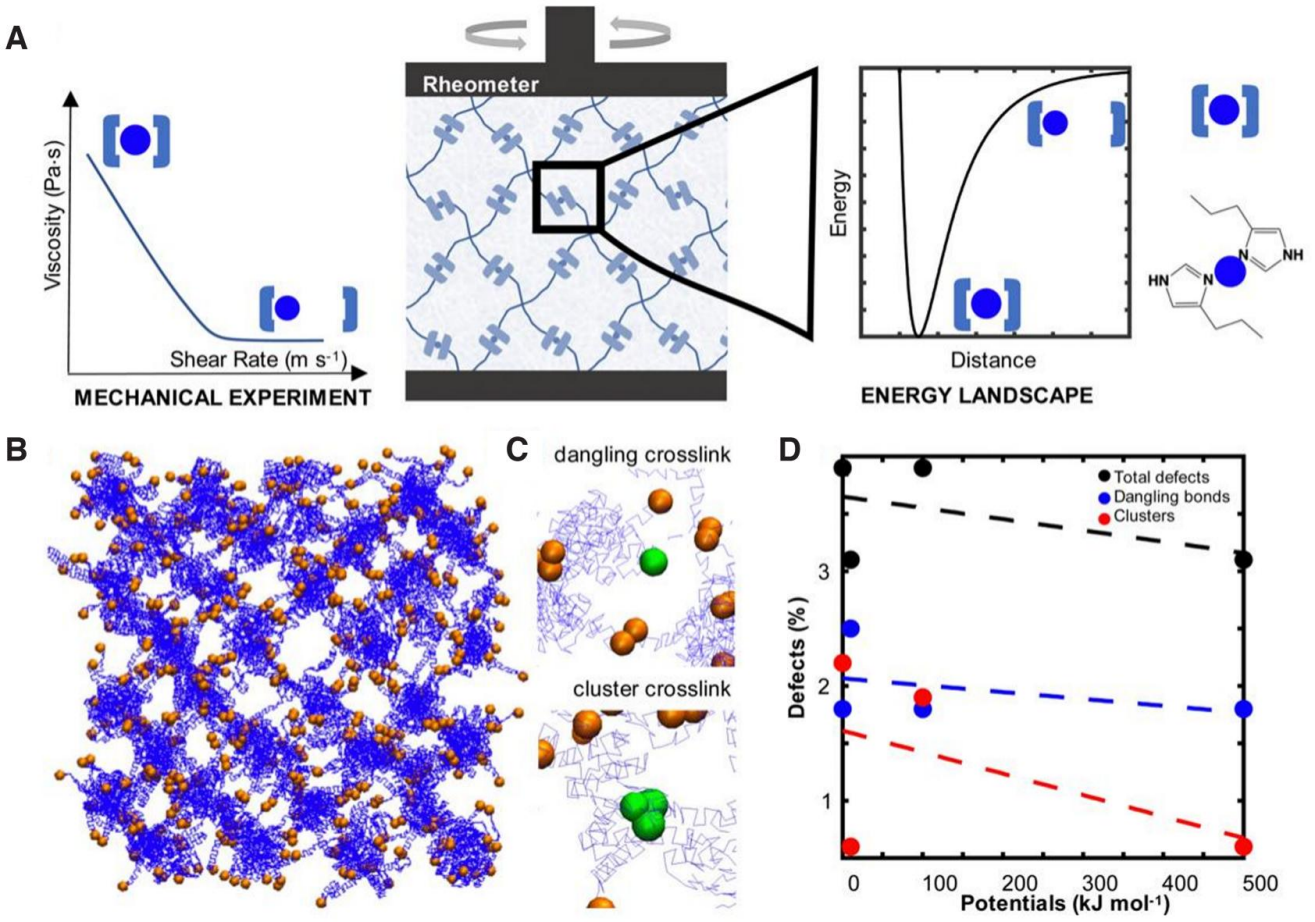
Polymer network setup. (**A**) Overview of simulation set up to probe the effect of crosslinker energy landscape on dynamic mechanical properties of ideal polymer hydrogel. The mechanical experiment measures the dynamic mechanical properties of an ideal metal-coordinated polymer network through a rheometer. The dynamic mechanical properties are related to the energy landscape of the crosslinker chemistry (metal–coordination bond). Based on early experimental works, 4-arm PEG–imidazole crosslinked with metal ions is represented. Here, the imidazole crosslinks via the nitrogen group to the Ni^2+^ metal ion in a metal–ligand 2 interaction. The network stoichiometry has a crosslinker/network functionality of 2, hence, the decision to model the network as pairwise interactions. While modeled based on the imidazole-family crosslinkers, the work here is widely applicable to several chemistries. (**B**) Equilibrated polymer network showing polymer strands (blue) and crosslinker beads (orange). The crosslinker beads are enlarged for clarity, and water beads are omitted. The polymer network is based on the crystal unit cell of an Ag_2_O tetrahedral crystal. (**C**) Examples of network defects, indicated by green markers, include dangling crosslinks (no bonding partner) and bond clusters (more than one bonding partner). The ideal network contains 2.7% defects based on individual bond crosslinks. (**D**) Percent of defects in network based on strength of interaction potential between crosslinking beads. Increasing the strength of the interaction potential reduces the total number of defects by reducing the number of dangling bonds present in the network. Reproduced with permission [173], Copyright 2024, Materials Advances.

The degree of crosslinking in a hydrogel has a very strong effect on the mechanical characteristics and adhesive behavior of the material, especially under wet conditions. It is necessary to increase the integrity of structure through prudent crosslinking to maximize the performance of hydrogel in diverse biological environments. As a rule, an increase in the crosslinking densities leads to increased mechanical strength and toughness. As Figure 8 shows, polymers with reversible crosslinks are dramatically stronger in toughness and self-healing [173]. In underwater assays, compressive strength may be optimized by varying the concentration of the components in hybrid hydrogel based on silk fibroin and chondroitin sulfate [174]. Hydrogels that are highly crosslinked are also highly adhesive, which is a vital feature in such applications like spinal dural repair. Crosslinked architecture with bioactive patches have high bursting pressure and are effective at keeping wet areas sealed [175]. Moreover, nanomaterials, such as GO incorporated into alginate-based hydrogels can also improve roles in biological environments [176]. Although high degrees of crosslinking density can greatly enhance the toughness and resilience of hydrogel in terms of strength [177], too much crosslinking would yield brittleness which can adversely affect functionality of a dynamic biological system. As a result, a balanced degree of crosslinking must be maintained between achieving maximum functionality without affecting the required mechanical compliance of the application [178].

#### Incorporation of conductive materials

**Figure 9:**
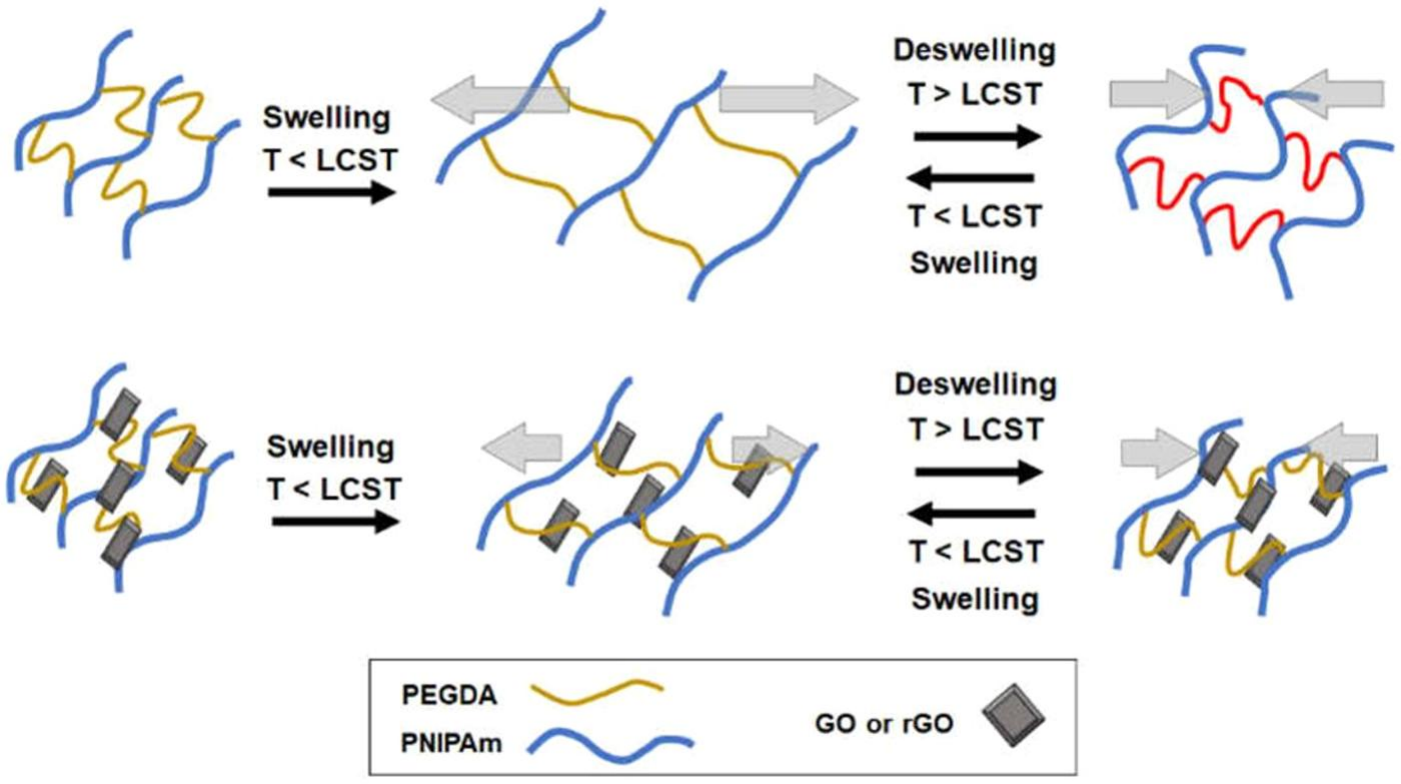
Schematic illustrations of controlling the thermoresponsive PNIPAM hydrogel actuators by forming a nanocomposite with GO or rGO. Reproduced with permission [179], Copyright 2022, *International Journal of Molecular Science*.

The inclusion of conductive materials, e.g. CNTs and GO, in the PNIPAM-based hydrogels has enhanced both their mechanical properties as well as functionality. For example, adding optimal amounts of CNTs to hydrogels can increase their toughness and boost electrical conductivity while maintaining desirable thixotropic properties and cytocompatibility, which are conducive to cell growth and proliferation [133]. Additionally, GO incorporated in hydrogel matrices has been shown to enhance generation of microcurrents, thus, aiding in cell division and cell growth and also reinforcing its critical role in tissue engineering use [132]. As illustrated in Figure 9, incorporation of GO or reduced GO (rGO) into PNIPAM hydrogel enables the precise control of mechanical and diffusional properties and, therefore, programmable actuation [179]. Dual cross-linking approaches additionally optimize local microarchitecture of such hydrogels, and lead to improved overall mechanical performance [180]. These advances explain why the presence of conductive substances does not only enhance PNIPAM-based hydrogels but also extends their use in the biomedical arena [173, 181].

Modification of polyaniline (PANI) and Ppy with PNIPAM provides a synergistic effect that significantly increases the mechanical properties of the composite material obtained. PANI, a material with high electrical conductivity and mechanical strength, increases the toughness and strength of PNIPAM, and PPy, which can release drugs controlled electronically, adds other functionality to the composite, especially drug delivery systems in biomedical applications [182, 183]. Introduction of these conducting polymers in PNIPAM hydrogels produces a stronger polymer network, which increases dynamic mechanical behavior and self-healing capabilities of PNIPAM hydrogels [173]. The optical characteristics of PANI and Ppy also provide the specific material with unique features that enhance the mechanical strength and responsiveness to external factors, which makes the composite material a high-quality substrate in advanced tissue engineering and drug delivery [183, 184].

Metal nanoparticles can also be used to strengthen and stabilize PNIPAM-based hydrogel in different environmental conditions. As an illustration, the production of magneto-controllable polymer microcarriers with the assistance of iron nanoparticles (FeNPs) in PNIPAM-co-acrylic acid (PNIPAM-PAA) microgels has displayed better colloidal stability and melodious to external magnetic fields which validates that such nanoparticles are intact under physiological conditions [185]. Introduction of silver nanowires into hydrogel composites is evaluated to have two advantages namely improved material qualities and intrinsic antimicrobial security in addition to capacity to analyze live feedback data in real time [51]. PNIPAM dressings with silver nanoparticles (AgNPs) are also expected to respond to temperature variation [186]. The use of multifunctional nanomaterials in the form of hydrogel networks provides better mechanical and thermoresponsive properties, which addresses the fundamental limitations of hydrogel networks like the absence of anti-tumor properties and behavior in bioimaging [187]. Metal complexation in supramolecular hydrogels has been demonstrated to provide soft, reversible architectures with viscoelastic characteristics suggesting that metal contacts can largely enhance the performance of hydrogel [177]. Moreover, both PANI and zinc oxide nanoparticles (ZnONPs) can be selected as surface functionalities that enhance the antibacterial quality and mechanical strength, which is essential to retain their condition in case of varying conditions [188]. Taken together, these results highlighted that metal nanoparticles have a lot of potential to enhance the permeability of PNIPAM-based hydrogels in a wide range of settings.

Altogether, the four reinforcement paths enhance PNIPAM-based dressing mechanics by achieving different yet complementary results. Hybrid nanocomposites enhance networks via providing more interaction and cross-linking sites (e.g. clay/CNT/GO/silica) with reported step-changes of mechanical metrics, such as *G*′ (MSNs: 1.3 ± 0.3 kPa to 32 ± 5 kPa) and compressive strength in silica-reinforced DN (0.10 MPa to 1.28 MPa). DN design obtains reinforcement through a rigid first network with a second network loosely cross-linked, able to retain integrity under repeated deformation and said to retain strong bonding both in dry and wet settings, which is accomplished through the joint, so-called, skeleton and surface adaptation roles of the two networks.

In comparison, dynamic crosslinking is placed as the pathway offering the greatest direct addition of self-healing, injectability and environmental adaptability, such as stiffness change to pH and salt variation; but its shortcoming lies in bond rupture, recreating structure at the cost of losing mechanical strength and excessive crosslinking produces brittleness. Lastly, conductive materials (CNTs/GO/rGO/PANI/Ppy) are discussed as reinforcing additives and simultaneously improving the mechanical properties, as well as broadening functionality (e.g. toughness and conductivity, microcurrent related bioeffects, programmable actuation, enhanced dynamic mechanical performance and self-healing), which makes them attractive in multifunctional systems. Nevertheless, the introduction of metal nanoparticles, CNTs or GO as a form of reinforcement introduces biocompatibility considerations that require careful control and optimization. These four mechanical reinforcement routes have been briefly compared in Table 4.

**Table 4:** Brief comparison of mechanical reinforcement routes discussed for PNIPAM-based hydrogel dressings.

Route	Main outcome	Key limitations	Reference
Hybrid nanocomposites	Strength↑ via added interaction and crosslink sites	Biocompatibility considerations; AgNP resistance, cytotoxicity risk	[160, 189]
DN hydrogels	Toughness/durability ↑ via rigid and soft network synergy; dry/wet adhesion	Complex and time-consuming	[105, 130, 135, 165,166]
Dynamic crosslinking	Self-healing, injectability, adaptable stiffness (pH/salt); toughness ↑	Self-healing occurs at the cost of losing mechanical strength; excessive crosslinking causes brittleness	[9, 131, 170, 178]
Conductive materials	Mechanical improvement, conductivity, self-healing functions	Potential biocompatibility risks if not optimized	[190]

### Approaches to enhance adhesion

Adhesion of PNIPAM-based wound dressings in dry environments can be enhanced via several surface modifications. Together, these modifications lead to improved performance of PNIPAM-based wound dressings.

#### Covalent/chemical coupling for wet adhesion

To improve adhesion, materials with a given wettability, and biomaterials with tailored surface topologies and chemical compositions have also been shown to be potentially useful in biomedical uses [191]. Surface modification with thiol (–SH) groups has been demonstrated to increase adhesion in wet environments through the disulfide bonding with tissue proteins. The adhesion of these bonds is strong in dynamic and moist environments, such as in wounds that are always exposed to fluids. Finally, the hydrogel is able to covalently bind to thiol groups on the tissue surface to form crosslinks with more functional groups to guarantee its stable fixation [192]. However, thiol functionalization can require additional processing to disclose –SH groups, and free thiols are prone to oxidation, therefore, S-protected or preactivated thiols are used to protect –SH groups and extend reactivity across a broader pH range [193]. The addition of CS to hydrogel-based liquid bandages can enhance adhesive, hemostatic and antibacterial properties, affecting the dressing’s performance when in contact with wet tissue [134].

#### Bioinspired surface chemistry modification

An example of one of the most successful surface treatments done to improve wet adhesion is based on the mussel adhesive proteins, which are catechol-based surfaces. Then the hydrogen bond and metal coordination bond form strong covalent and noncovalent bonds between dry and wet surfaces and catechol. However, once used with PNIPAM-based hydrogels, catechol groups enhance the capacity of a hydrogel to adhere to moist, uneven or even submerged surfaces by strongly bonding with the tissue in a chemical way [194]. This modification helps overcome the hydration layer that typically weakens adhesion in aqueous environments [195]. In addition, catechol incorporation in PNIPAM hydrogels has been reported to allow temperature-controlled reversible adhesion, and microscopic channels can facilitate water removal from the interface to further enhance adhesive strength [45].

It is notable that catechol functionalization can reduce the hydrogel integrity, which can impact adhesive performance and stability [196]. Thus, the catechol substitution degree and the hydrogel structure design play a crucial role in ensuring that the limitations related are surpassed and maximum performance of biomedical use is reached [195, 197]. This modification comes in handy especially in wet surroundings in which conventional adhesive mechanisms often do not take hold. The hydrogel may be attached to the tissue by catechol groups to enhance initial attachment and also under physiological conditions, long term adhesion.

#### Surface activation and grafting methods

The physical technique of altering the surface chemistry of PNIPAM-based hydrogels includes plasma treatment and grafting under ultraviolet (UV) light. Plasma treatment aids the addition of functional groups to the surface of the hydrogel, which is an improvement to wettability and adhesion [198]. In addition, plasma treatment can make naturally hydrophobic surfaces hydrophilic, and this also enhances the performance of adhesives [199]. It has been reported that atmospheric pressure plasma treatments (APPTs) offer multiple benefits over conventional corona treatment regarding the ability to control the physicochemical characteristics of surface modification [200]. However, the major weakness is hydrophobic recovery whereby the surface that was treated takes a long time to restore the original hydrophobic nature, thus, creating a problem of long-term adhesive stability [199]. Further tricks have been documented to stabilize the increased wettability and adhesion by crosslinking the surfaces and demonstrate the need to reduce hydrophobic recovery to maintain long term adhesive effects [201].

The surface can be UV grafted with bioactive molecules or adhesion-promoters to increase the contact between the hydrogel and the biological tissue [202]. UV grafting has also been reported to enhance the adhesion on both dry and wet surfaces by the formation of covalent bonds between the hydrogel structure and the surface. Such a method enhances surface hydrophilicity and lowers contact angles and helps with the enhanced adhesion on various substrates [203, 204]. Also, light-induced grafting depends on a surface-linked initiating species, and identifying appropriate anchor moieties to be covalently attached is still a significant issue [205].

Also, by grafting biodegradable polymers like poly(3-hydroxybutyrate-co-3-hydroxyvalerate), surface pullulan is achieved to have increased biocompatibility and a more functioning surface that enhances interaction with biological tissue [206].

#### Interfacial coatings and multilayer engineering

A layer-by-layer (LbL) method is applied through the successive deposition of alternating polymers having antagonistic charges onto the hydrogel surface. This strategy produces a multilayered surface which can be well adjusted to enhance the adhesion properties. They may be made up of polyelectrolytes, proteomes or peptides that may be designed to bond to the tissue surfaces either by electrostatic or hydrogen bonding or van der Waals forces in augmenting adhesion under either dry or wet conditions. In addition to that, wound dressing behavior can be enhanced further by the addition of bioactive molecules such as growth factors and antimicrobial agents into LbL [207].

**Figure 10:**
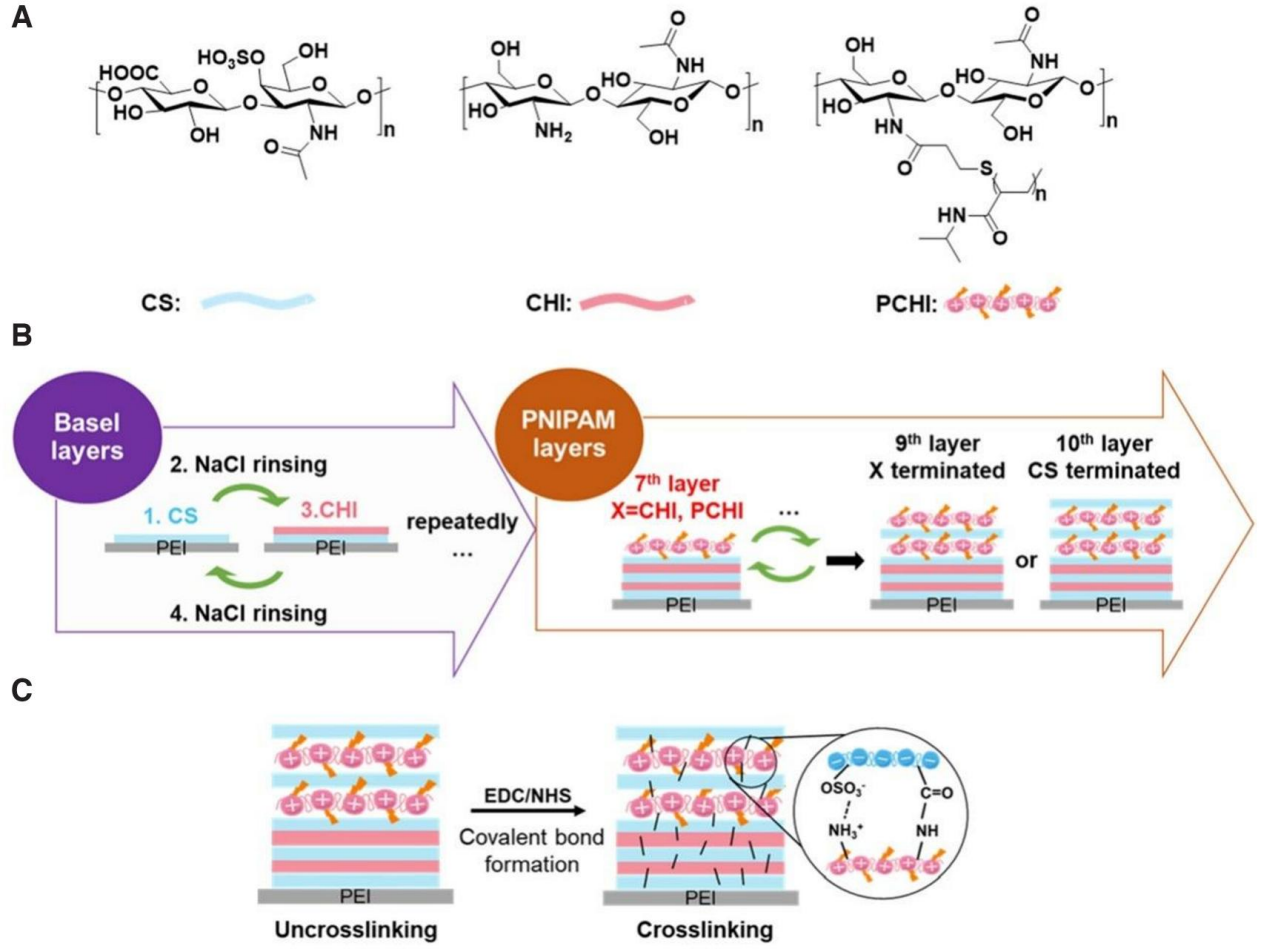
(**A**) Molecular units of polymers used in the polyelectrolyte multilayer (PEM) buildup: chondroitin sulfate (CS) as polyanion, chitosan (CHI) and PNIPAM-grafted-chitosan (PCHI) as polycations. (**B**) Schematic representation for preparation of PCHI−PEM using the layer-by-layer (LbL) technique. The substrate was coated with positively charged polyethylene imine (PEI) as an anchoring layer followed by subsequent CS and CHI adsorption. CHI was replaced by PCHI from 7th layer. (**C**) Crosslinking of the PEM using EDC/NHS to form amide bonds between CHI or PCHI and CS. Reproduced with permission [208], Copyright 2023, Chinese Roots Global Impact.

Regarding the PNIPAM hydrogels, the LbL method will allow the introduction of bioactive substances that will provide better bioactivity and adhesion of the hydrogel, which is displayed in Figure 10. These multilayers can have temperature responsive swelling and deswelling characteristics offered by the thermoresponsive property of PNIPAM, and this is important in regulating the wettability and adsorption of proteins onto the surface to enhance cell adherence. It is also possible with the LbL assembly process to incorporate crosslinking agents that further stabilize the multilayers and provide mechanical strength to the multilayers, and therefore, they can be used in biomedical applications (tissue engineering, etc.) [208].

However, problems persist regarding the industrial portability of the LbL process, including the fluid and quality of raw materials especially in the case of using expensive biological materials, and the amount of time to produce the film, particularly with dip-coating experiments. Further, a more fundamental understanding and careful characterization of LbL systems is urgently needed; biopolymer-based LbL films are quite challenging to traditional electron microscopy or X-ray characterization, which is to great part due to their high-water content, organic structure and nanometer-thickness [207]. Moreover, interfacial chemistry under soft multilayer surfaces by means of surfactants promotes the formation of a continuous, strong interface, which enhances the total durability and mechanical strength of dressings in wet conditions [209].

Silane coupling agents can be grafted onto the surface of PNIPAM-based hydrogels to improve adhesion to various surfaces. Specificity of covalent bonds is formed between silane agents and the matrix of the hydrogel and target tissue, leading to a stronger and more durable interface. In wet environments, these agents stabilize the adhesive strength by forming a stable siloxane network that resists hydrolysis and retains adhesive properties. Such modification is suitable for producing a hydrophilic surface enabling water molecules to spread over the hydrogel surface, and thus, contact tissues closely, thereby enhancing adhesion in both dry and wet conditions [210]. However, limitations may include the need for optimal ratios of silane to polymer, as excessive cross-linking can lead to reduced flexibility and potential brittleness, affecting the overall performance of hydrogel dressings in clinical applications [211].

Another commonly used surface modification to improve both biocompatibility and adhesion in the wet is by using PEG. PEG coatings reduce protein adsorption and biofouling and reduce protein adhesion to tissues without triggering an immune response. Moreover, the hydrophilicity of PEG keeps the hydrogel hydrated, with the wound environment moist and conducive to healing. In addition, PEG prevents desiccation of hydrogel in dry environments, allowing it to remain adhesive for longer periods [212], and due to its low surface energy, it also maintains adhesion to wet surfaces. However, the degradation of PEG-based adhesives can affect mechanical properties, where a decrease in storage modulus is observed due to the presence of elastically ineffective PEG polymers trapped within the network, leading to reduced adhesion over time [213].

Across these methods, catechol and thiol systems are considered to be the best in the wet environment because of their ability to undergo a strong chemical anchorage onto the moist tissues, which include irregular and submerging conditions in catechol cross-linking and thiol disulfide cross-linking respectively. On the contrary, strategies driven by plasma or UV irradiation mainly enhance adhesion by altering surface properties, namely, adding functional groups, increasing surface energy and enhancing hydrophilicity, to enable enhanced contact on dry and moist tissues. Interfacial coatings focus on interface control, including tunable multilayers with LbL and bioactive incorporation, PEG for preventing protein adsorption and biofouling while maintaining hydration, and silane to stabilize the interface, so they serve best when interfacial stability requires longer periods and surface-function design. In Table 5, a brief comparison of these adhesion-reinforcement strategies is provided.

**Table 5:** Comparative summary of adhesion enhancement routes.

Route	Advantages	Limitation	Reference
Catechol functionalization	Stronger adhesion on moist/uneven/submerged tissue; improves initial and long-term adhesion	Mechanical property changes	[194, 196]
Thiol functionalization	Wet adhesion via disulfide bonding with tissue proteins	Require additional processing, free thiols prone to oxidation	[192, 193]
Physical surface activation/grafting (Plasma + UV)	Increase surface functional groups, surface energy, hydrophilicity, promote contact on dry and wet surfaces	Hydrophobic recovery and reduced long-term effect over time for plasma	[198, 199, 202]
Interfacial coatings/multilayer engineering (LbL + PEG + silane)	LbL: Tunable multilayer interface, incorporate bioactive PEG: Reduced protein adsorption Silane: Strong interfacial stability	Scale up, time cost, materials cost, quality issues, characterization challenges	[207, 210–213]

With these modifications, PNIPAM-based hydrogels can gain the mechanical strength necessary to be used in many underwater environments, and then, be used in advanced wound dressing applications.

## Translational challenges and limitations of PNIPAM-based hydrogel dressings for regenerative medicine

PNIPAM has gained immense research attention due to its thermoresponsive properties and physiologically acceptable transition temperature making it applicable to drug delivery and tissue engineering. However, PNIPAM displays a significantly low level of biodegradability in physiological environment, which causes some concerns about its long-term application *in vivo* and its possible bioaccumulation [214]. Biodegradable-based copolymerization with poly(D, L-lactide) (PLA) was shown to allow degradation of the hydrogel mass through hydrolysis of PLA, but the segments of PNIPAM themselves are not degradable [27]. Dual-responsive and naturally modified PNIPAM systems have been considered as well, but the main objective of these strategies is to maintain thermoresponsiveness or to improve mechanical and biocompatibility properties instead of improving PNIPAM biodegradability [215, 216]. Consequently, PNIPAM is inherently nonbiodegradable in physiological environments, and thus, poses a persistent challenge for its long-term biomedical application [214, 215].

Beyond biodegradability concerns, PNIPAM hydrogels have some other weaknesses concerning their interaction with biological conditions and their thermoresponsive behavior. PNIPAM hydrogels have been associated with potential inflammatory responses due to immune system interactions and strategies have been implemented to counter this inflammatory interaction, including the introduction of anti-inflammatory agents or the creation of systems that release drugs upon detection of an inflammatory signal [217]. Simultaneously, LCST hysteresis, defined by the difference between swelling and deswelling transition temperatures, may also affect hydrogel behavior and is controlled by polymer composition and cross-linking density, which are often adjusted to achieve better responsiveness and mechanical [140]. Besides, the mechanochemical properties of PNIPAM hydrogels are also fundamental in drug delivery constructs and can likewise be manipulated via structural engineering, e.g. ushering in some other monomers or IPNs [28, 218]. All these factors combined to highlight the inherent relationship between PNIPAM hydrogel functioning, immune response and thermoresponsiveness, where extreme care must be taken to attain effective biomedical applications.

To address the shortcomings of mechanical and functional capabilities of PNIPAM-based hydrogel wound dressings, the latter are typically reinforced with metal nanoparticles (e.g. CNTs or GO), nevertheless, such reinforcements also create more biocompatibility issues. Specifically, the application of metal nanoparticles and, particularly, AgNPs has become a cause of concern because of the development of bacterial resistance and possible cytotoxicity, thus, necessitating a tight regulation of concentration and usage conditions [189]. In the same way, nanomaterials that are carbon-based, such as CNTs and GO, may accumulate unexpected biocompatibility risks by disrupting the processes of wound healing and tissue regeneration by responding negatively or by causing inflammation unless designed optimally [190]. In spite of the fact that nanoparticle reinforced hydrogels confer to multifunctional wound dressings increased mechanical efficiency and regenerative capacity [47], large-scale *in vivo* and *in vitro* testing is mandatory to reduce the risk of biocompatibility and guarantee safe clinical use [189, 190].

Clinical translation of thermoresponsive hydrogel wound dressing is faced with considerable challenges because of the challenge of environmental instability and practical limitations. The temperature oscillations that cause changes in performance and drug release characteristics breed environmental uncertainties within such systems [219]. The ever-changing microenvironment of a dynamic wound bed may undermine the efficacy of hydrogel, whereas mechanical performance metrics like adhesion and stretchability are important when dealing with mobile body parts, as structural integrity may be compromised with repetitive motion [16, 220]. Another limitation in clinical translation is the need to have precise control over the thermoresponsive behavior to address the practical guidelines involved in the preparation and delivery of injectable hydrogels [221].

In general, the clinical translation of PNIPAM-based hydrogels is constrained by correlative material, biological and application-level limitations. The clear recognition of these issues provides an important conceptual basis on the intelligent development of new generation thermoresponsive wound dressing.

## Clinical relevance and wound-specific design considerations of PNIPAM dressings

The clinical usefulness of PNIPAM-based wound dressings is directly linked to the ability to customize the materials design to suit the critical needs of the different wound types.

1. PNIPAM-based have been widely studied to treat diabetic wounds due to ability to react to changes in body temperature [46]. Dual crosslinked PNIPAM systems, including networks with Gum Arabic, offer controlled bioactive release, improved mechanical strength and tissue adhesion, thus, offering antibacterial and anti-inflammatory properties that lead to wound healing [22]. Self-healing PNIPAM copolymer hydrogels grafted with gallic acid possessing a reactive oxygen species scavenging property have also been shown to have enhanced tissue adhesion, antibacterial and greatly enhanced diabetic wound healing *in vivo* [222]. Other advanced designs like 4D printed PNIPAM-based hydrogels with curcumin-loaded micelles and degradable crosslinkers also have potential in enhancing wound healing and tissue repair in diabetic models [223].
2. PNIPAM-based hydrogels have also been extensively used in the management of burn wounds adapting to the body temperature whilst providing mechanical strength and flexibility [47]. Functional PNIPAM composites, such as PNIPAM/GO-Ag hydrogel, have been demonstrated to possess reduced inflammation, greater collagen deposition and greater fibroblast growth factor expression, which accelerate healing in deep second degree burn models [224]. PNIPAM hydrogel incorporating pH-responsive silk fibroin/sodium alginate nanoparticles has facilitated the controlled release of antibiotics and growth factors, facilitated re-epithelialization and inhibited infection rates in severe burn wounds [225].
3. PNIPAM-based hydrogels show significant potential in treatment of wounds located on the mobile regions of the body. Dual-crosslinked PNIPAM constructs increase mechanical strength, adhesion in tissues and bioactivity, which in turn sustain stable performance during repetitive deformation, and therefore, make them a viable option when using them on wounds in constant motion [16, 22]. Multifunctional PNIPAM hydrogel patches have demonstrated reversible adhesion through contraction-relaxation transitions, fatigue resistance and self-healing properties, thus, making it easy to attach and work when in a dynamic state [226]. In addition, the mechano-responsive characteristics of PNIPAM hydrogels provide possibility to release drugs under mechanical pressure, which provides long-term therapeutic results in highly mobile wound locations [20].
4. PNIPAM-hydrogels, in contracted close to body temperature, assist in controlling excessive exudate [227]. Composite PNIPAM systems that incorporate hydrophilic polymers, like alginate, have shown greater uptake of fluid and water retention as compared to pure PNIPAM, thus, making them better at trapping wound exudates [228]. PNIPAM hydrogels swelling kinetics can be adjusted using network composition enabling exudate regulation without distorting wound moisture which is beneficial for healing. Although the absorption capacity inherent in PNIPAM alone might be lower than some dressings exhibiting high levels of absorption, PNIPAM composites demonstrate meaningful fluid handling potential [229].

Traditional wound dressings, including alginate, hydrocolloid and polyurethane (PU) foam dressings are commonly used in clinical settings and have a role in established standards of care in defined wound conditions, as mentioned in the recent systematic reviews. These dressings primarily provide passive wound management functions, by retaining moisture, absorbing exudates or mechanically cushioning wounds, but do not quickly respond to dynamics of the wound microenvironment [230–232]. Whereas, hydrogel-based wound dressings, particularly PNIPAM-based systems, have functionally adaptive properties, such as thermoresponsiveness, tunable adhesion and controlled drug delivery [20, 28]. A comparative overview is provided in Table 6.

**Table 6:** Clinical comparison of conventional wound dressings (alginate, hydrocolloid, polyurethane foam) and hydrogel-based systems, highlighting functional characteristics, clinical applications and limitations.

Dressing type	Clinical role	Advantages	Limitations	Reference
Alginate	Widely used for clinical wound management	High exudate absorption; gel formation upon contact with wound fluid; suitable for moist wound environments	Limited suitability for dry wounds; primarily passive function without responsiveness	[230, 233]
Hydrocolloid	Common clinical dressings for chronic wounds and moisture management	Occlusive coverage; maintenance of moist wound environment; support for autolytic debridement	Limited absorption capacity; not suitable for wounds with high exudate	[231]
PU foam	Standard clinical dressings for exudative wounds requiring cushioning and protection	Good fluid absorption; mechanical protection; thermal insulation	Lack of active or responsive functionality; limited adaptability to wound microenvironment changes	[232]
Hydrogel-based	Increasingly studied as advanced wound dressings in recent literature	Moisture regulation; conformability to wound bed; compatibility with functional modification	Lower absorption capacity compared to alginate or foam when used alone	[20, 231]
PNIPAM-based	Investigated as functionally adaptive hydrogel systems for wound care	Thermoresponsive behavior; tunable physical properties; potential for controlled release and adaptive performance	Clinical translation challenges; need for formulation optimization and validation	[20, 28]

## Conclusion and outlook

The hydrogel dressings based on PNIPAM possess unique capabilities in comparison to soft wound care products as their LCST, close to body temperature, allows easy application, quick *in vivo* gelation and adhesion to irregular wound surfaces. In the literature, the primary emphasis is placed on the fact that clinically useful performance cannot be defined as a single strong property, but rather as the combination of bulk mechanics, interfacial adhesion, hydration control and time dependent viscoelastic dissipation.

Mechanical reinforcement techniques such as hybrid nanocomposites, DN designs and dynamic or sacrificial crosslinking enhance toughness and stretchability, and the advantages of these techniques can be applied to wound sealing only when the interface does not degrade in the presence of wet conditions and under repeated motion. Similarly, the adhesive designs which are proven to be effective on dry surfaces tend to lose their strength when interfacial water exists, which is why wet adhesion can and must depend not only on proper surface chemistry but also on energy loss in bulk. These areas are additionally related by rheological behavior: shear-thinning and recovery control injectability and shape retention, and moduli change with frequency/amplitude control adhesiveness and failure mode during deformation of real tissues.

Despite the rapid developments, there are several knowledge gaps, which cause hindrances in cross-study comparison and slow translation. To start with, mechanical and adhesive metrics are frequently reported at inconsistent conditions like substrate type, hydration degree, temperature relative to LCST, loading rate and test geometry, hence, making it difficult to benchmark. Second, adhesion is often represented as a single peak value and other cyclic fatigue, long-term stability in exudate and interfacial failure mapping are less frequently reported. Third, rheology is not always coupled with adhesion results, although the effect of viscoelastic dissipation on debonding and injectability is very strong. Fourth, concerns of biocompatibility and durability can be dealt with later in the development process especially when dealing with systems that include nanoparticles, conductive fillers or reactive functional groups.

Future research would benefit a more standardized and clinically realistic design and testing framework. Temperature (in relation to LCST), hydration condition and time after application should be reported along with monotonic and cyclic adhesion tests on biologically relevant tissues. Rheological characterization must extend beyond a single *G*′ value to include sweeps of amplitude and frequency, yield behavior, thixotropy/recovery and relationship to application modality, i.e. injectable, printable or patch. At the materials level, the focus should be on degradable PNIPAM copolymer and bioresorbable cross-link chemistries to deal with persistence issues and interface design should focus on wet adhesion by combined approaches that control interfacial water and augment bulk dissipation. Finally, the need to align with sterilization requirements, scalable manufacture, and *in vivo* models to capture wound movement, exudate, and a healing stage is also needed in translation. These directions hold the potential to transform PNIPAM dressings out of promising laboratory formulations into robust, clinically relevant systems with repeatable behavior and better-understood regulatory interactions.
